# The Gated Cascade Diffusion Model: An Integrated Theory of Decision Making, Motor Preparation, and Motor Execution

**DOI:** 10.1037/rev0000464

**Published:** 2024-07

**Authors:** Edouard Dendauw, Nathan J. Evans, Gordon D. Logan, Emmanuel Haffen, Djamila Bennabi, Thibault Gajdos, Mathieu Servant

**Affiliations:** 1 Laboratoire de recherches Intégratives en Neurosciences et psychologie Cognitive, INSERM, Université de Franche-Comté; 2 Maison des Sciences de l’Homme et de l’Environnement, CNRS, Université de Franche-Comté; 3 Department of Psychology, Ludwig Maximilian University of Munich; 4 Department of Psychological Sciences, Vanderbilt University; 5 Centre de Recherche en Psychologie et Neuroscience, CNRS, Aix-Marseille Université

**Keywords:** Decision-making, Motor preparation, Motor execution, Diffusion model, Kalman-Bucy filter

## Abstract

This article introduces an integrated and biologically inspired theory of decision making, motor preparation, and motor execution. The theory is formalized as an extension of the diffusion model, in which diffusive accumulated evidence from the decision-making process is continuously conveyed to motor areas of the brain that prepare the response, where it is smoothed by a mechanism that approximates a Kalman–Bucy filter. The resulting motor preparation variable is gated prior to reaching agonist muscles until it exceeds a particular level of activation. We tested this gated cascade diffusion model by continuously probing the electrical activity of the response agonists through electromyography in four choice tasks that span a variety of domains in cognitive sciences, namely motion perception, numerical cognition, recognition memory, and lexical knowledge. The model provided a good quantitative account of behavioral and electromyographic data and systematically outperformed previous models. This work represents an advance in the integration of processes involved in simple decisions and sheds new light on the interplay between decision and motor systems.

## Introduction

Many of our internal choices are communicated to the world, and this communication requires an interplay between decision and motor systems. For instance, the choice to vote for a candidate in an election eventually results in the deposit of a ballot in a box (or pushing a button on a voting machine). Deciding who to have friendships and relationships with results in concrete approach/avoidance behaviors. Choices about where to spend our money determine our consumer behavior. Decision and motor systems are also jointly engaged in many experimental cognitive tasks. For instance, recognition memory tasks, lexical decision tasks, perceptual decision tasks, numerosity judgment tasks, and conflict tasks all involve a decision between two or more options (e.g., old/new, greater/less than a quantity), each option being mapped to a specific motor plan (e.g., manual button press, saccade toward a target, vocal response). Decision and motor systems have each benefited from extensive research (for reviews, see Cisek and Kalaska, 2010; Ebbesen and Brecht, 2017; Forstmann et al., 2016; Freedman and Assad, 2016; Gold and Shadlen, 2007; Lemon, 2008; O’Connell and Kelly, 2021; Ratcliff and Smith, 2004; Ratcliff et al., 2016; Robinson, 1973; Schall, 2019; Schall and Paré, 2021; Summerfield and Parpart, 2022), and recent modeling efforts have sought to specify the relationship between them (Servant et al., 2015, 2021; Verdonck et al., 2021). However, as will become obvious in the next sections, current models fail to capture important aspects of empirical data, either at the motor preparation or at the motor execution processing levels. The present work aims to address these short-comings by introducing an integrated theory of decision-making, motor preparation, and motor execution. The theory builds upon a gated cascade evidence accumulation architecture and incorporates a filtering mechanism at the motor preparation level, for which we provide a computational foundation.

The article is structured as follows. We will first review traditional theoretical conceptions regarding the relationship between decision and motor stages and recent neurophysiological data that challenge them. We will then highlight the shortcomings of current modeling approaches and introduce the integrated theory.

### Deciding and Acting: Traditional Views and Challenges

A traditional view in psychology is that the motor system is engaged when the decision-maker has committed to an internal choice (Donders, 1969; Logan and Cowan, 1984; Sternberg, 1969). The decision process produces a discrete result, indicating which response to prepare and execute. This view still persists in contemporary decision-making models, according to which noisy evidence from our senses and memory is accumulated until a threshold quantity of cumulative evidence is attained (e.g., Bogacz et al., 2006; Evans and Wagenmakers, 2020; Laming, 1968; Ratcliff and Smith, 2004; Tillman et al., 2020). Each accumulator is associated with a specific choice, and the accumulator that first reaches the threshold determines the choice and the duration of decision formation. The choice is then passed on to the motor system and does not carry any information about the strength of the evidence.

A growing body of neurophysiological evidence challenges this traditional view however. Electroencephalographic (EEG) studies have identified two electrical signals that exhibit key signatures of the theoretical accumulation-to-threshold decision variable (for reviews, see O’Connell and Kelly, 2021; O’Connell et al., 2018). The first signal, termed centroparietal positivity (CPP), reflects accumulated sensory evidence and culminates to a threshold voltage around the time of the response. The CPP appears whenever an individual has to make a decision between two options and shows the same temporal dynamics and spatial topography regardless of sensory and response modalities. Importantly, the CPP appears even when participants are instructed to make the decision mentally (*i*.*e*., without communicating the outcome through the motor system; O’Connell et al., 2012) or when the stimulus-response mapping is not yet known during stimulus presentation (Twomey et al., 2016). Although the precise functional interpretation of the CPP requires additional investigations (O’Connell and Kelly, 2021), these empirical findings suggest that it may reflect a decision about alternative categories of a stimulus with a neural generator in the parietal cortex.

The second signal corresponds to effector-selective motor preparation EEG activities (de Jong et al., 1988; Gratton et al., 1988; Pfurtscheller and Lopes da Silva, 1999), such as the lateralized readiness potential or the decrease in spectral activity in the mu/beta band over the motor cortex (in case of left/right manual responses). Similar to the CPP, effector-selective EEG signals appear to reflect the theoretical accumulation-to-threshold decision variable. Although ramping electrical activities of the two classes of signals overlap in time and reach their voltage peak around the time of the response, the onset latency of effector-selective signals occurs after the onset latency of the CPP (Kelly and O’Connell, 2013). In addition, effector-selective EEG signals are absent when participants are instructed to make the decision mentally or when the stimulus-response mapping is not yet known during stimulus presentation (O’Connell et al., 2012; Twomey et al., 2015). These results suggest that when decisions are mapped onto actions, the decision variable is represented in motor areas of the brain that prepare the response. Similar findings have been observed using magnetoencephalography (de Lange et al., 2013; Donner et al., 2009), functional resonance imaging (Filimon et al., 2013; Tosoni et al., 2008), transcranial magnetic stimulation (Klein-Flügge and Bestmann, 2012), and single-unit recordings in awake monkeys (Gold and Shadlen, 2000, 2003, 2007; Purcell et al., 2010; Ratcliff et al., 2003; Schall, 2019).

**Figure 1:**
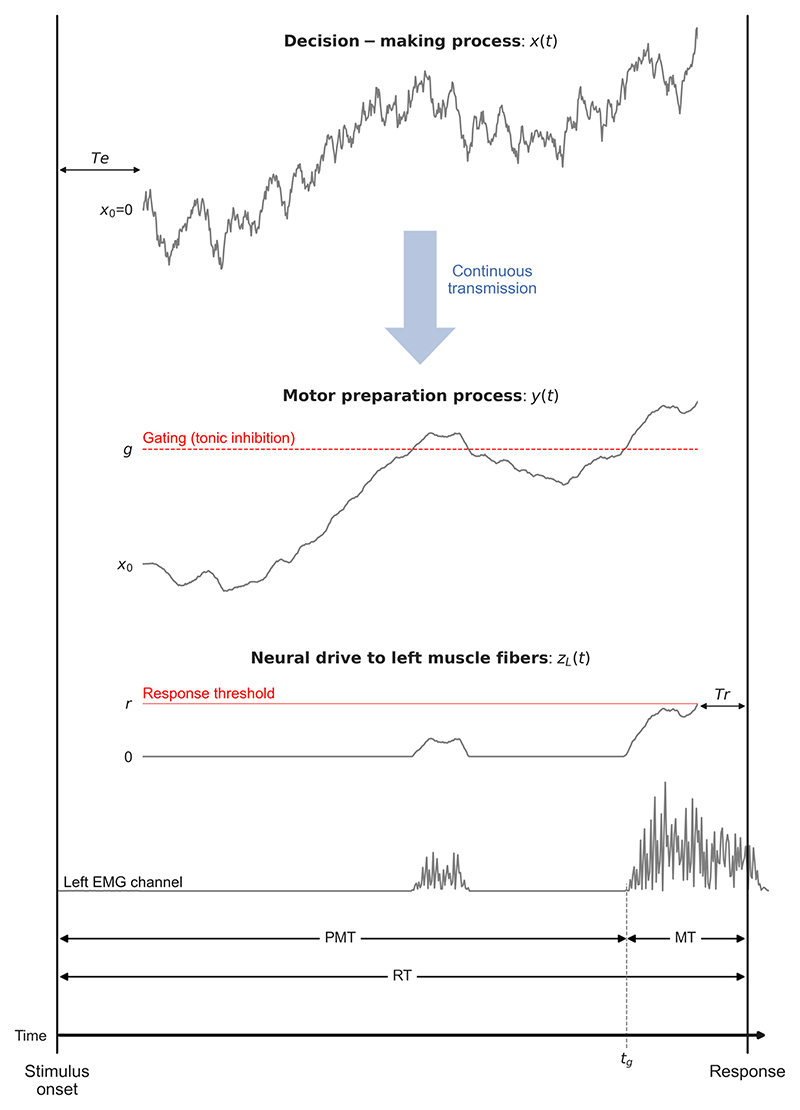
Architecture of the gated cascade diffusion model with a filtering mechanism at the motor preparation level (GCDF) *Note*. In this trial, the model predicts a right response and a partial EMG burst in the right EMG channel. See text for details. EMG = electromyographic; PMT = premotor time; MT = motor time; RT = reaction time. See the online article for the color version of this figure.

Another source of neurophysiological evidence that speaks against strict serial discrete processing between decision and motor stages comes from surface electromyographic (EMG) studies. EMG is a non-invasive technique that measures the electrical activity of muscles through electrodes placed on the skin surface. Recording the EMG activity of agonist muscles in a reaction time (RT) task (e.g., the *flexor pollicis brevis* for a button press with the thumb) allows researchers to partition each RT into two latencies: a premotor time (PMT, from stimulus onset to the EMG onset of the response; see Figure 1) and a motor time (MT, from EMG onset to the response; Botwinick and Thompson, 1966; Weiss, 1965). Recent studies have shown that both mean PMT and mean MT increase as the perceptual discriminability of the stimulus decreases (Servant et al., 2021; Weindel et al., 2021; see also Selen et al., 2012, for similar findings obtained with a different EMG methodology based on reflex gains). These results demonstrate that EMG activity reflects a quantity that scales with sensory evidence and suggest a flow of the decision variable down to agonist muscles. The flow is not purely continuous because EMG bursts have a discrete onset (that occurs ~150-180 ms on average before the response for a button press with the thumb, with ~900 gram-force required; see Servant et al., 2021).

### Modeling the Interplay Between Decision and Motor Systems

Servant et al. (2021, 2015) proposed a dual-threshold diffusion model to account for the aforementioned neurophysiological findings, with a particular focus on EMG findings. The theory concerns two-choice decisions that are mapped onto actions and assumes that the decision variable is continuously transmitted to motor areas of the brain that prepare the response (such as premotor and primary motor cortices for body movements). Through continuous flow, some of the work usually done in forming motor commands can be done during decision formation, providing an advantage in terms of processing time (Eriksen and Schultz, 1979; McClelland, 1979; Shadlen and Kiani, 2013). In addition, this architecture offers substantial flexibility to motor control by allowing for real-time modulations and revisions of evolving motor commands based on incoming evidence (Nakayama et al., 2023; Resulaj et al., 2009; Stone et al., 2022). This flexibility appears particularly important in real-life settings, as individuals are constantly dealing with a changing environment. For instance, the affordance competition hypothesis states that the brain continuously processes sensory information to determine an ensemble of possible actions while simultaneously gathering information to select among these actions (Cisek, 2007).

The theory further assumes that the transmission of information from motor preparation to agonist muscles is continuous but regulated by a gate. The gate determines the minimum level of accumulated evidence required to pass on the decision variable to muscle fibers and is presumably mediated by the basal ganglia system (hypothesized to act as a gate-keeping mechanism for the execution of motor plans; e.g., Frank, 2011; Hikosaka, 2007; Mink, 1996). The gate might serve two main purposes. First, it prevents low levels of accumulated evidence from exciting muscle fibers. Low levels of accumulated evidence are associated with a low likelihood that the decision is correct, so gating these activations prevents unnecessary muscular activity. Second, the gate offers a shield against unwanted behaviors. These purposes are consistent with the gating function of the basal ganglia system: Patients with basal ganglia disorders often encounter difficulties initiating purposeful movements and exhibit involuntary movements such as tremors and chorea (Hikosaka, 2007; Mink, 1996).

Formally, the decision variable follows a diffusion process (Ratcliff, 1978):

(1) $$dx(t)=vdt+\sigma dW(t),\;x(0)=x_{0},$$

where *x*(*t*), *v, σ*, and *W*(*t*), respectively, correspond to the accumulated evidence at time *t*, the drift rate, the diffusion coefficient, and a Brownian motion. Parameter *x*_0_ represents the starting point of the process. If there is no bias for a particular response, *x*_0_ = 0.

The gate was originally formalized as a threshold (termed "EMG threshold") superimposed on *x*(*t*). Here, we propose a different interpretation (though mathematically equivalent) of the gate as a constant inhibition. This interpretation is consistent with the tonic inhibitory control of the basal ganglia over motor areas (Hikosaka, 2007; Mink, 1996) and allows for a clearer description of inputs to muscle fibers. Specifically, in the context of a choice task involving left versus right manual responses, inputs to left and right muscle fibers, variables *z*_*L*_(*t*) and *z*_*R*_(*t*), respectively, are defined as follows:

(2) $$\{\begin{array}{l} z_{L}(t)\;=\max(-x(t)-g,0)\\ z_{R}(t)\;=\max(x(t)-g,0), \end{array}$$

where parameter *g* corresponds to gating inhibition. Variables *z*_*L*_(*t*) and *z*_*R*_(*t*) are classically referred to as neural drive. The electrical excitation of muscle fibers, measured by EMG, starts when the neural drive becomes positive. The full-wave rectified EMG signal^1^ can be interpreted as a noisy approximation of the neural drive to the area of muscle over which the electrodes are placed (Dideriksen and Farina, 2019; Farina et al., 2010; Vigotsky et al., 2018). For this reason, we will represent full-wave rectified EMG signals (instead of raw signals) in the present work.

Researchers in biomechanics have evidenced a strong association between the neural drive, the resulting electrical excitation of muscle fibers, and force production (for a review, see Vigotsky et al., 2018).^2^ Consequently, the model assumes that the response (e.g., a button press) is issued when a particular level of neural drive has been produced, which is modeled by applying a threshold (parameter *r*) on *z*_*L*_(*t*) and *z*_*R*_(*t*). A left response is issued if variable *z*_*L*_(*t*) first reaches *r*, whereas a right response is issued if variable *z*_*R*_(*t*) first reaches *r*. We refer to *r* as response threshold. This parameter depends on the force required to give the response, properties of agonist muscles, and muscular variables specific to each individual (e.g., strength and endurance).

This processing architecture makes EMG predictions (such as distributions of PMT and MT) that are strictly identical to the dual-threshold diffusion model proposed by Servant et al. (2021, 2015). The only difference concerns the interpretation of the gate as a constant inhibition (instead of a threshold), which translates into a minor parametric change^3^ and prompts a renaming of the model. We refer to it as *gated cascade diffusion model* (GCD) to emphasize the main processing components (diffusion decision variable, continuous flow, and gate).

In each trial, the predicted PMT corresponds to the latency between the onset of evidence accumulation and the time at which *z*_*L*_(*t*) or *z*_*R*_(*t*) becomes positive. Note, however, that the latter event can occur more than one time during a trial due to noisy fluctuations of the accumulated evidence variable *x*(*t*) around the gate. This phenomenon allows the model to predict a well-established EMG phenomenon termed partial EMG burst (e.g., Coles et al., 1985; Servant et al., 2015). A partial EMG burst corresponds to a small burst of muscular excitation that sometimes occurs during PMT and that does not generate sufficient force to issue the response (for empirical illustrations, see Appendix A). The predicted PMT thus corresponds to the latency between accumulation onset and the time *t*_*g*_ at which *z*_*L*_(*t*) or *z*_*R*_(*t*) becomes positive for the last time before reaching the response threshold, consistent with the empirical definition (Figure 1). The predicted MT corresponds to the latency at which *z*_*L*_(*t*) or *z*_*R*_(*t*) hits the response threshold minus *t*_*g*_. In addition, predicted PMT and MT each incorporate residual processing components with mean duration *Te* and *Tr*, respectively. At minimum, *Te* includes stimulus encoding processes and *Tr* includes the electromechanical delay (time lag between muscle excitation and a measurable change in force output). This delay involves both electrochemical and mechanical processes (e.g., propagation of action potentials, excitation-contraction coupling, force transmission along the active and passive parts of the series elastic component; Cavanagh and Komi, 1979). In its raw form (without between-trial variability in any of the model components and with an unbiased starting point of evidence accumulation *x*_0_ = 0), GCD has five free parameters: drift rate *v*, gating inhibition *g*, response threshold *r*, and mean residual latencies *Te* and *Tr*.

Because muscular excitation is determined by *x*(*t*), modulations of drift rate impact both predicted PMT and MT. Consequently, GCD predicts an increase in mean PMT and mean MT as the perceptual discriminability of the stimulus decreases, explaining empirical EMG findings reported in the previous section. Servant et al. (2021) derived other predictions from the model. First, for any given drift rate, distributions of PMT and MT should exhibit a similar right-skewed shape, which should translate into an approximately linear PMT quantile-MT quantile plot. Second, and as mentioned before, GCD sometimes predicts partial EMG bursts during PMT. The proportion of trials containing at least one partial burst and the latency of the first partial burst should increase as the drift rate decreases. Third, for any given drift rate, the between-trial correlation between PMT and MT should be null, due to the Markov property of the diffusion process (given the present, the future does not depend on the past).

To test these predictions, Servant et al. (2021) recorded the EMG activity of muscles associated with left/right manual responses in a random dot motion task. In each trial, participants had to determine the global direction (left vs. right) of moving dots and press the corresponding response button with their left or right thumb. The proportion *p* of dots moving coherently in the left or right signal direction, termed motion coherence, was manipulated across six levels (*p* = 0, .05, .08, .12, .2, .4), in order to modulate the perceptual difficulty of the decision. The EMG data provided evidence for each prediction.^4^ In addition, fits of GCD to the joint distributions of PMT and MT in correct and incorrect trials and to accuracy data were good, providing quantitative evidence for the model.

**Figure 2:**
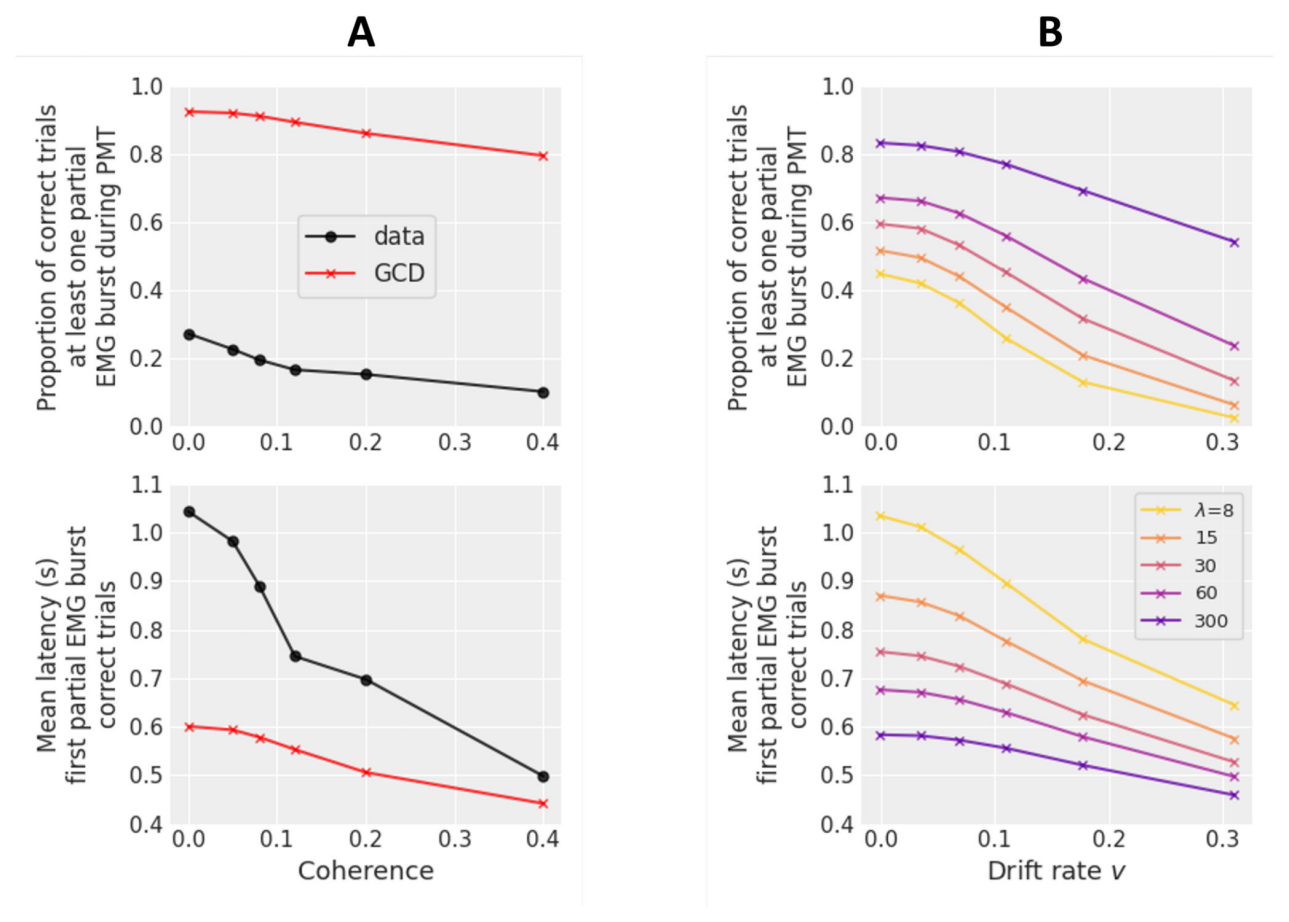
Partial EMG Burst Statistics in a Random Dot Motion Task With Varying Levels of Motion Coherence and Model Predictions *Note*. (A) Proportion of correct trials containing at least one partial EMG burst during PMT (upper plot) and mean latency of the first partial burst (lower plot) averaged across subjects as a function of motion coherence. Observed data are shown as black dots, and GCD predictions are shown as red crosses. (B) GCDF simulations, with varying levels of leak λ and drift rate *v*. Apart from the leak parameter, simulations used the best fitting GCD parameters averaged across subjects reported by Servant et al. (2021) and 100,000 simulated trials per condition. GCD = gated cascade diffusion model; GCDF = gated cascade diffusion model with a filtering mechanism at the motor preparation level; PMT = premotor time; EMG = electromyographic. See the online article for the color version of this figure.

However, Servant et al. (2021) did not attempt to fit the proportion and latency of partial EMG bursts, nor did they examine the predictive accuracy of the model with respect to these variables. Figure 2A shows the observed versus predicted proportion of correct trials containing at least one partial EMG burst during PMT (upper plot) and the mean latency of the first partial EMG burst (lower plot) averaged across subjects for each motion coherence condition. Model predictions are computed using the best fitting parameters from Servant et al. (2021) and 100,000 simulated trials per condition. GCD strongly overestimates the proportion of correct trials containing at least one partial burst during PMT and underestimates the average latency of the first partial burst, especially for low coherence levels. Similar results were obtained when considering both correct and incorrect trials and by taking the median latency of the first partial burst. These results demonstrate that the amount of within-trial noise predicted by GCD is too large, causing too many oscillations around the gate. To solve this problem, one may be tempted to decrease the diffusion coefficient (parameter *σ* in Equation 1) that regulates the amplitude of within-trial noise. However, the diffusion coefficient is fixed at an arbitrary value to satisfy a scaling property within the model (Ratcliff, 1978). This means that adjusting the value of *σ* –either to a different fixed value or as a free parameter– has no impact on model predictions, as any modulation will be counteracted by a proportional modulation of the other model parameters to produce identical predictions. This analysis suggests that a processing step is missing in GCD.

As reviewed in the previous section, EEG studies have identified two electrical signals that exhibit key signatures of the theoretical accumulation-to-threshold decision variable, with important functional differences. The first signal (the CPP) appears to perform a decision about alternative categories of a stimulus and is fully supramodal. We refer to this processing stage as *decision making*. The second signal corresponds to effector-selective *motor preparation* activities. Decision making and motor preparation EEG signals also exhibit a different sensitivity to strategic influences, as manipulations of response bias and speed-accuracy modulate motor preparation signals but not the CPP (Kelly et al., 2021; Steinemann et al., 2018). GCD approximates decision making and motor preparation by a single evidence accumulation diffusion process, but this assumption does not capture the lag between the two corresponding EEG signals, nor does it capture their anatomical and functional differences. The same criticism applies to the diffusion model (Ratcliff, 1978) or to other evidence accumulation models such as the leaky competing accumulator (Usher and McClelland, 2001), the linear ballistic accumulator (Brown and Heathcote, 2008), racing diffusion models (Ratcliff et al., 2003; Tillman et al., 2020), and Poisson counter models (Ratcliff and Smith, 2004; Vickers, 1970).

Verdonck et al. (2021) recently hypothesized that the evidence accumulation decision variable is continuously transmitted to motor areas of the brain that prepare the response, similar to Servant et al. (2021, 2015). They further assumed that the decision variable is filtered during motor preparation. Formally, the evidence accumulation variable *x*(*t*) follows a diffusion process identical to Equation 1. The motor preparation variable *y*(*t*) takes *x*(*t*) as a continuous input and performs a leaky accumulation according to the following differential equation:

(3) $$dy(t)=\lambda (x(t)-y(t))dt,\;y(0)=x_{0},$$

where *λ >* 0 corresponds to the leak parameter. The response is executed when *y*(*t*) reaches one of two thresholds. As can be seen by solving Equation 3, the motor preparation variable is obtained by applying a smoothing filter to *x*(*t*):

(4) $$y(t)=\lambda \int_{-\infty }^{t}dt^{\prime }x\left(t^{\prime }\right)e^{\lambda \left(t^{\prime }-t\right)}.$$

The value of the decision variable at *t − t′* seconds before the current time *t* contributes *x*(*t′*)*e*^*λ*(*t′−t*)^ to the value of the motor preparation variable at time *t*. The motor preparation variable *y*(*t*) thus corresponds to the weighted sum of past states *x*(*t′*) of the decision-making variable, with weights exponentially decreasing at rate *λ* (the leak parameter). When *λ* approaches infinity (high leak), all weights on past states tend to zero, and the model reduces to the diffusion model (Ratcliff, 1978). Conversely, as *λ* decreases (low leak), the number of past states of the decision-making variable contributing to the motor preparation variable increases. This results in a reduction of random noise. The amount of smoothing at the motor preparation level thus increases as *λ* decreases. Verdonck et al. (2021) further showed that for large values of *t*, the mean of *y*(*t*) is delayed by *λ*^*−*1^ relative to the mean of *x*(*t*), corresponding to the filter-related delay. A modulation of *λ* thus produces a modulation of the speed and accuracy of the decision.

This cascade evidence accumulation architecture for decision making and motor preparation, termed leaky integrated threshold model (LIT) by the authors, provides a straightforward explanation to the partial temporal overlap between corresponding neurophysiological activities, their rise-to-threshold morphology, and the modulation of their respective accumulation rate by stimulus difficulty. It also allows for specific strategic influences at each processing stage. Although Verdonck et al. (2021) did not test LIT against neurophysiological data, they showed that it provides a better account of behavioral data than the diffusion decision model in three data sets (a face/car discrimination task, a lexical decision task, and a random dot motion task). Two of these data sets included a speed-accuracy manipulation, which was better explained by a variation of leakage than the variation of decision thresholds commonly assumed in the literature.

There are two theoretical issues with the LIT framework. First, the rationale for the model is not clear. The diffusion model is known to implement an optimal strategy in the sense that it minimizes the expected decision time for a given expected accuracy level (Bogacz et al., 2006). Consequently, it is not clear why the motor preparation process would average past accumulated evidence (containing less information) with current accumulated evidence (containing more information). The second issue concerns motor execution. If the threshold operates at the motor preparation level, as in the original model definition, the choice is categorically communicated to the muscles for execution, and the model cannot account for the modulation of mean MT by stimulus discriminability and partial EMG bursts. Alternatively, one may assume that the threshold operates at the motor execution level and corresponds to the response. This hypothesis, however, would lead to a continuous activation of response-relevant muscles, at odds with EMG bursts. The integrated theory of decision making, motor preparation, and motor execution introduced in the next section provides a solution to both problems. We further illustrate how the theory can capture partial EMG burst statistics.

### An Integrated Theory of Decision Making, Motor Preparation, and Motor Execution

We first propose a computational foundation (Marr, 1982) for the motor preparation process. The brain is a noisy information processing system (Shadlen and Newsome, 1994), and the decision variable is likely corrupted by noise during its continuous transmission to motor preparation areas. Therefore, an important goal of motor preparation might be to recover the original decision variable from noise. Formally, the corrupted decision variable $\tilde{x}$ received by the motor preparation process can be defined as:

(5) $$\tilde{x}(t)=x(t)+\xi U(t),\;\tilde{x}(0)=x_{0},$$

where *ξU* (*t*) corresponds to white Gaussian noise with standard deviation *ξ* added during the transmission process. It is well known that the Kalman-Bucy filter provides the optimal solution to this problem in the sense that the Kalman-Bucy filtered process $\tilde{y}$ minimizes the mean squared prediction error $E\left[{\left(x(t)-\tilde{y}(t)\right)}^{2}\right]$ (Kalman and Bucy, 1961; Øksendal, 2003). $\tilde{y}$ satisfies the following differential equation (see Appendix B, for the mathematical derivation):

(6) $$d\tilde{y}(t)=\frac{\sigma }{\xi }\tanh\left(\frac{\sigma }{\xi }t\right)(\tilde{x}(t)-\tilde{y}(t))dt+vdt,\;\tilde{y}(0)=x_{0}.$$

The term $\frac{\sigma }{\xi }$ tanh $\left(\frac{\sigma }{\xi }t\right)$ corresponds to the so-called Kalman gain and determines the amount of smoothing of $\tilde{x}(t)$ needed to optimally recover *x*(*t*) from noise.

Equation 6 poses an important challenge for the motor preparation system: it requires knowledge of the ratio between *σ* (diffusion coefficient) and *ξ* (amplitude of transmission noise) as well as the drift rate *v*, which is implausible. After all, if the motor preparation system had knowledge of the drift rate, no decision-making mechanism would be necessary. Consequently, these parameters may be replaced by priors under strategic control. Let parameter *λ*’ denotes the prior on the ratio between *σ* and *ξ*, and parameter *v′* the prior on the drift rate. If there is no bias toward a particular stimulus/response, a reasonable prior for *v′* is *v′* = 0. Equation 6 thus becomes:

(7) $$d\tilde{y}(t)=\lambda^{\prime }\tanh\left(\lambda^{\prime }t\right)(\tilde{x}(t)-\tilde{y}(t))dt,\;\tilde{y}(0)=x_{0},$$

Equation 7 can be viewed as the best estimate of the decision variable *x*(*t*) that the motor preparation system can plausibly provide, given available information.^5^ It turns out the motor preparation process *y*(*t*) from LIT is very similar to $\tilde{y}(t)$. Note that the gain *λ′* tanh (*λ′t*) quickly increases to its asymptotic value *λ′*, and therefore:

(8) $$d\tilde{y}(t)\approx \lambda^{\prime }(\tilde{x}(t)-\tilde{y}(t))dt,\;\tilde{y}(0)=x_{0}.$$

Comparing Equations 3 and 8, the main difference between $\tilde{y}(t)$ and *y*(*t*) concerns the input to motor preparation: $\tilde{y}(t)$ takes the corrupted decision variable $\tilde{x}(t)$ as input, instead of the decision variable *x*(*t*) (see Appendix B, for additional mathematical details). The prior *λ′* corresponds to the leak parameter *λ* and can be interpreted in a similar way. To summarize, the filter at the motor preparation level can be viewed as an attempt to recover the original decision variable from noise. This mechanism has an important consequence that will become clear in the next section: it prevents unnecessary muscular activity triggered by random noise in the accumulated evidence and thus appears complementary to the action of the gate in stabilizing motor control.

The above analysis suggests that a complete theory of decision making, motor preparation, and motor execution may require a combination of GCD and LIT models. This combination can still be viewed as a gated cascade diffusion model, but we add the letter F at the end of the acronym of the model to indicate that the decision variable is filtered during motor preparation (GCDF). An illustration of GCDF is provided in Figure 1. Decision making and motor preparation are modeled by *x*(*t*) (Equation 1) and *y*(*t*) (Equation 3), respectively. We chose *y*(*t*) instead of $\tilde{y}(t)$ to save one free parameter (the amplitude of transmission noise *ξ*) and reduce the risk of trade-offs between parameters. This choice does not have any impact on our main conclusions, since model variants incorporating *y*(*t*) versus $\tilde{y}(t)$ provide a comparable fit performance to data (see Appendix C), consistent with the above analysis. Gating inhibition now operates on *y*(*t*), so inputs to left and right muscle fibers, variables *z*_*L*_(*t*) and *z*_*R*_(*t*), respectively, are defined as follows:

(9) $$\{\begin{array}{l} z_{L}(t)\;=\max(-y(t)-g,0)\\ z_{R}(t)\;=\max(y(t)-g,0). \end{array}$$

The response (left vs. right) is determined by the variable that first hits the response threshold *r*, similar to GCD.

### Model Simulations

Within GCDF, the smoothing mechanism at the motor preparation level should reduce the predicted proportion of partial EMG bursts and increase their mean latency (due to the filter-related delay). Figure 2B shows simulations of the model with varying levels of leak *λ* and drift rate *v*. Similar to GCD, the model predicts an increase in the proportion of correct trials containing at least one partial EMG burst during PMT (upper plot) and an increase in the average latency of the first partial burst as the drift rate decreases (lower plot). Importantly, and as predicted, the former decreases and the latter increases as *λ* decreases. Additional analyses of simulated data showed that GCDF predicts an increase in mean PMT and mean MT as drift rate decreases for each level of leak, and an approximately linear PMT quantile-MT quantile plot. Interestingly, low leak levels produce a small positive between-trial correlation between PMT and MT, especially for high drift rates (Figure D1). This complex pattern results from two opposite forces: the Markov property of the diffusion process on the one hand (that predicts a null correlation between PMT and MT) and the filtering process on the other hand (that reduces random fluctuations and positively increases the correlation). Because processing components are likely variable from trial to trial (e.g., Laming, 1968; Ratcliff and Rouder, 1998), we explored the effect of between-trial variability in GCDF parameters. The only noticeable difference in model predictions was caused by between-trial variability in drift rate (normally distributed with mean *v* and standard deviation *sv*). This source of variability produces a positive between-trial correlation between PMT and MT, especially for low leak and high drift rate levels (Figure D2). It also predicts a slightly curvilinear PMT quantile-MT quantile plot (the departure from linearity increases as *sv* increases).

Given the complexity of GCDF, it is difficult to guarantee that these predictions are robust across the whole (plausible) parameter space. In our opinion, a complete test of the model requires three key ingredients: (a) a quantitative fit to both behavioral and electrophysiological data (EEG and EMG); (b) a comparison with GCD as a benchmark using model selection techniques; and (c) an evaluation of the fit quality of the model to both electrophysiological and behavioral data from a range of choice RT tasks that tap into different cognitive domains. The latter is important because it will offer an assessment of the generality of the model and delineate potential boundary conditions of application. We aimed to incorporate the three ingredients in the present work in order to provide the first attempt to jointly model decision making, motor preparation, and motor execution processing stages. However, we restricted our analyses to behavioral and EMG data from choice tasks that tap into four different cognitive domains (motion perception, numerical cognition, recognition memory, and lexical knowledge) in order to maintain a manageable amount of electrophysiological and modeling work. Although EEG data could supplement our assessment of decision making and motor preparation processes, the poor signal-to-noise ratio of EEG forces researchers to apply a low-pass filter to smooth the signal, obscuring the degree to which the brain may perform such filtering.

## Experiment 1: Motion Perception

As a first step, we fit GCDF to the joint distributions of PMT and MT in correct and incorrect trials and to accuracy data from the left/right random dot motion task of Servant et al. (2021). Following Palmer et al. (2005), we assumed a linear relationship between motion coherence and drift rate. We predicted a better performance (balance between fit quality and parsimony) of GCDF compared to GCD, especially with regard to partial EMG burst statistics. Arguably, there are several ways to incorporate partial burst statistics into the loss function, quantifying the discrepancy between data and model predictions. We chose the following scheme for its simplicity. Accuracy data were divided into six trial types: pure- correct trial (pureC: correct response, no partial EMG burst during PMT), correct-correct trial (CC: correct response, at least one partial EMG burst during PMT, first partial burst located in the correct EMG channel), incorrect-correct trial (IC: correct response, at least one partial EMG burst during PMT, first partial burst located in the incorrect EMG channel), and so forth for incorrect responses (pure-incorrect trial pureI, incorrect-incorrect trial II, correct-incorrect trial CI). The proportion of each of these six trial types was incorporated into the loss function. Comparisons between GCD and GCDF were performed with and without between-trial variability in processing components in order to examine the robustness of findings.

### Method

Critical details of the experiment are presented below, but readers are directed to Servant et al. (2021) for full details. Eighteen healthy and right-handed participants (two men; age range: 18-32; mean age: 21.1) from the University of Franche-Comté performed a random dot motion task with six levels of coherence (0, .05, .08, .12, .2, .4). In each trial, participants had to determine the global direction (leftward vs. rightward) of dots and press the corresponding response button with their left or right thumb. The sampling rate of the response device was 1,000 Hz, and the force required to press each button was ~900 gram-force. The EMG activity of response agonists (the *flexor pollicis brevis* in particular) was recorded by means of two electrodes fixed 1 cm apart on the skin of the thenar eminence of each hand. Participants performed 12 blocks of 96 trials each, with a short break between blocks. Within each block, trials were defined by a factorial combination of motion direction (left vs. right) and motion coherence (six levels). All types of trials occurred equally often and were presented in a random order. Each trial started with the presentation of the random dot motion stimulus, which remained on the screen until the participant responded. An RT deadline was set to 5 s, and the interval between the response to the stimulus and the next trial was 1.5 s.

Bipolar EMG signals (sampling rate = 1,024 Hz) were high-pass filtered using a 10 Hz cut-off (3^*rd*^ order Butterworth filter) and epoched -0.5 s to 5 s relative to stimulus onset. For each epoch, EMG burst onsets were detected using a three-step semi-automatic procedure (see Servant et al., 2021). Trials with a high level of noise were discarded from analyses (7.5% of trials on average; range 0.2%-24%).

#### Models and Fit Procedure

GCD and GCDF were coded in C, using the method and framework of Evans (2019). The fit procedure was coded in Python. The time step was set to .001 s to provide the same granularity as the behavioral and EMG data, and the diffusion coefficient *σ* (see Equation 1) was fixed at .1 to satisfy a scaling property within the models (see general introduction). Following Servant et al. (2021), we fixed the starting point of the decision-making process at *x*_0_ = 0, since left and right responses were equiprobable.^6^ Consequently, we modeled incorrect/correct responses instead of left/right responses (with negative evidence favoring the incorrect response and positive evidence favoring the correct response). In its raw form (i.e., without between-trial variability in any of the model parameters), GCDF has six free parameters: the slope *k* of the linear relationship relating motion coherence to drift rate, gating inhibition *g*, response threshold *r*, mean residual latencies *Te* and *Tr*, and the leak parameter *λ*. The raw GCD has five free parameters (all GCDF parameters except *λ*), and the full GCD has four additional parameters (between-trial variability in drift rate *sv*, starting point *sx*_0_, and residual latencies *sTe* and *sTr*). *sv* corresponds to the standard deviation of a Gaussian distribution with mean *v. sx*_0_, *sTe*, and *sTr* correspond to the range of a uniform distribution with mean *x*_0_, *Te*, and *Tr*, respectively. These distributional assumptions are directly inherited from standard applications of the diffusion model (Boehm et al., 2018; Ratcliff and Rouder, 1998; Voss et al., 2004; Wiecki et al., 2013). The full GCDF has one additional between-trial variability parameter, corresponding to between-trial variability in leakage (uniformly distributed with range *sλ* and mean *λ*). A uniform distribution was chosen because we do not have any theoretical assumption about the distributional shape of variability in leakage. All free parameters were constrained to be *≥*0 and were not allowed to vary between motion coherence conditions. Parameters *sx*_0_, *sTe, sTr*, and *s*λ were further constrained to not exceed 180% of *g, Te, Tr*, and λ, respectively. The models were fit to each individual data set by minimizing the following loss function (likelihood-ratio chi-square statistic):

(10) $$G^{2}=2\sum_{i=1}^{6}\sum_{j=1}^{6}\sum_{k=1}^{6}\sum_{l=1}^{6}n_{ijkl}\text{log}\left(\frac{n_{ijkl}/n_{i}}{n_{ijkl}^{\prime }/n_{i}^{\prime }}\right).$$

Summations over *i* and *j* extend over the six motion coherence levels and the six trial types (pureC, CC, IC, pureI, II, CI; see the Introduction section of this experiment), respectively. Summations over *k* and *l* extend over the six bins bounded by PMT quantiles (.1, .3, .5, .7, and .9) and the six bins bounded by MT quantiles (.1, .3, .5, .7, and .9) respectively.^7^ The variables *n*_*ijkl*_ and $n_{ijkl}^{\prime }$ refer to the observed and simulated number of trials in coherence condition *i*, trial type *j*, PMT bin *k*, and MT bin *l*. Finally, the variables *n*_*i*_ and $n_{i}^{\prime }$ refer to the observed and simulated number of trials in coherence condition *i*, and *log* refers to the natural logarithm. The *G*^2^ statistic thus characterizes the goodness-of-fit of the model to the joint distributions of PMT and MT and to the proportion of each of the six trial types. It was minimized using differential evolution (Storn and Price, 1997) and 20,000 simulated trials per condition. Observe that we did not incorporate the latency of partial EMG bursts into the *G*^2^ formula in order to mitigate the potential impact of artifactual partial bursts on the fit quality of other aspects of the data. The latency of partial bursts can thus be considered as out-of-sample data, and the comparison between these data and model predictions will serve as a generalization test of the models.

Before turning to model comparison techniques, it is important to note that GCD is nested in GCDF: the two models are equivalent when the leak parameter λ approaches infinity. Consequently, a low best fitting leak value would indicate that GCDF adds to a GCD description of the data. The key question is whether this improvement in fit quality is sufficiently important to justify the additional complexity of GCDF. To answer this question, the *G*^2^ was converted to both Akaike information criterion (AIC) and Bayesian information criterion (BIC):

(11) $$AIC=G^{2}+2m,$$

(12) $$BIC=G^{2}+m\text{log}(N),\;$$

where *m* corresponds to the number of free parameters, *log* corresponds to natural logarithm, and *N* equals the number of observations used in the *G*^2^ computation. BIC and AIC thus both penalize for model complexity but in a different way. Since both statistics have advantages and drawbacks (Vrieze, 2012), we report both of them, hoping for consistency between model decisions. For each individual subject, the best model is the one associated with the smallest AIC or BIC. If 14 (or more) out of 18 subjects support one model over the other in terms of AIC or BIC (two-sided binomial test), then the result is significant.

## Results

**Figure 3:**
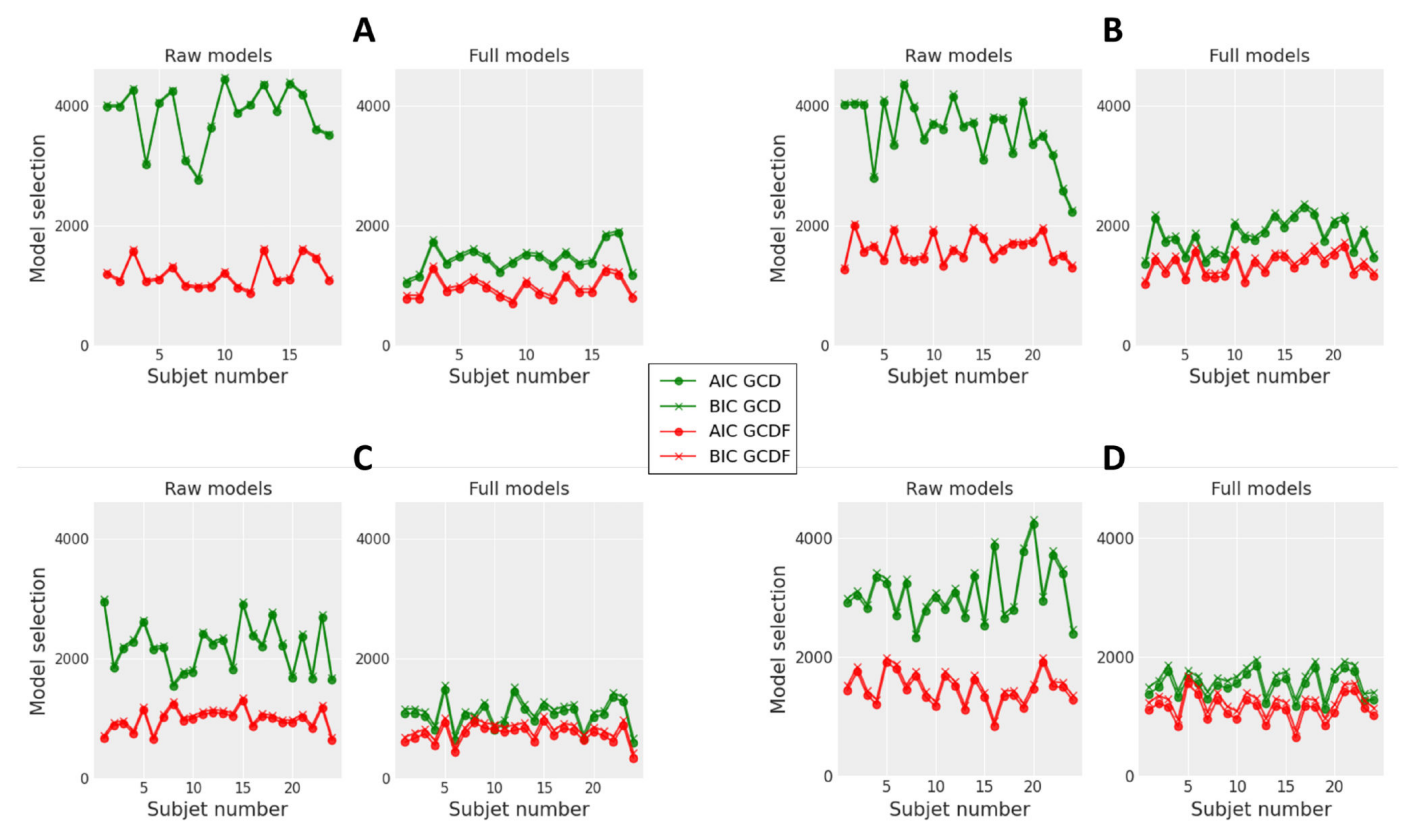
Model Selection Statistics for Experiments 1–4 *Note*. Panels (A)–(D) correspond to Experiments 1–4. AIC = Akaike information criterion; BIC = Bayesian information criterion; GCD = gated cascade diffusion model; GCDF = gated cascade diffusion model with a filtering mechanism at the motor preparation level. See the online article for the color version of this figure.

GCDF was associated with lower AIC and BIC statistics compared to GCD for each of the 18 subjects of the experiment and for both raw and full model variants (Figure 3A). The difference in AIC and BIC between raw and full models was much smaller for GCDF compared to GCD, indicating that between-trial variability in GCDF parameters has a minor impact on model performance, contrary to GCD. In fact, the raw GCDF was associated with lower AIC (BIC) statistics compared to the full GCD for 16 (16) subjects. This analysis provides strong evidence for the superiority of GCDF.

**Figure 4:**
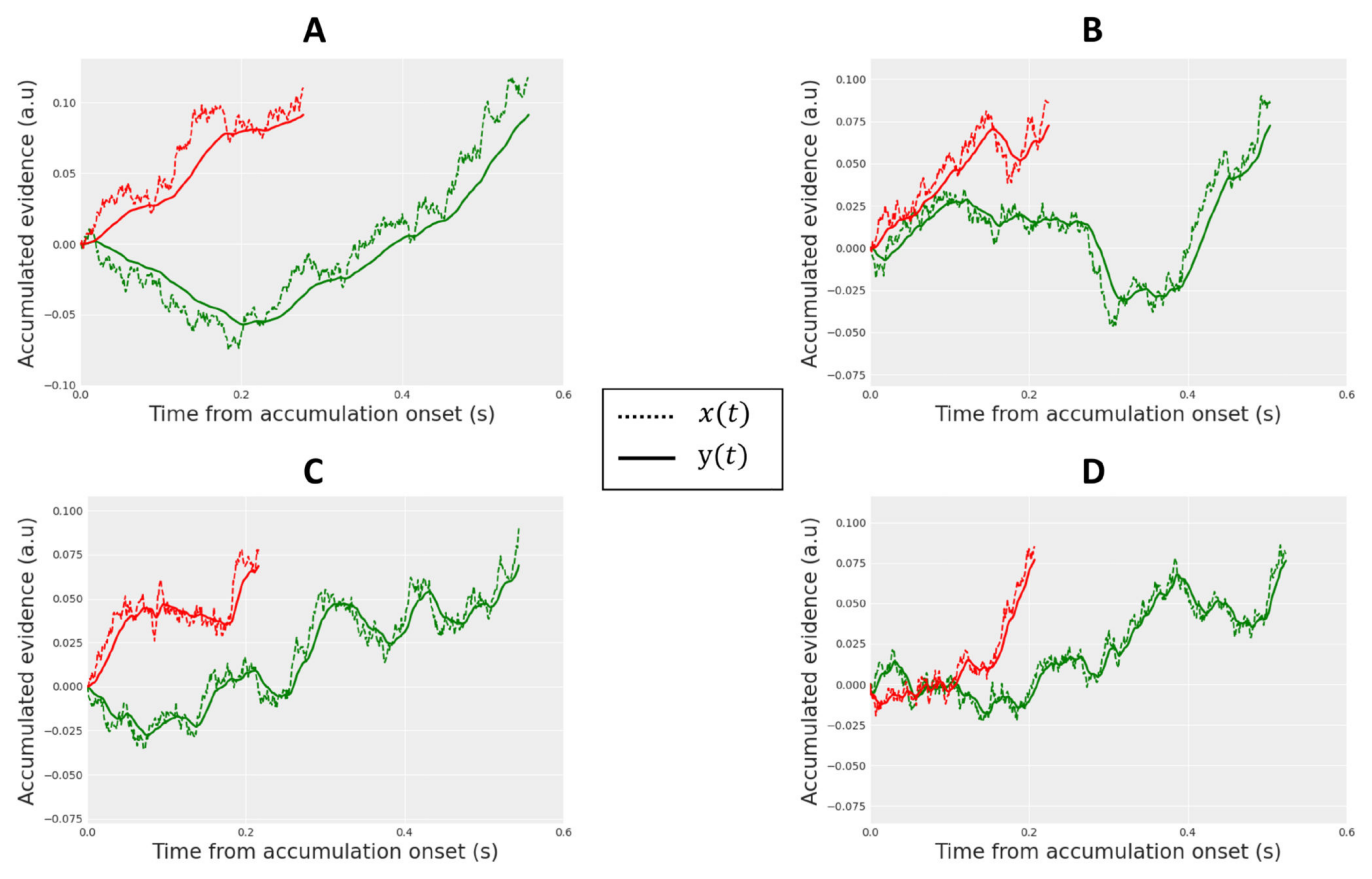
Trajectories of Decision Making *x*(*t*) and Motor Preparation *y*(*t*) Variables Computed From the Full GCDF Using Best Fitting Parameters Averaged Across Subjects From Experiments 1–4

*Note*. Panels (A)–(D) correspond to Experiments 1–4. For each experiment, two correct trials (red and green trajectories) were simulated using best fitting parameters from the full GCDF averaged across subjects, with the constraint of giving latencies between accumulation onset and response threshold crossing in range .2–.3 s (red trajectory) and .5–.6 s (green trajectory) to facilitate comparison between experiments. Between-trial variability parameters were set to 0, and drift rate corresponded to condition coherence = 8% for Experiment 1, mean numerosity = 58 for Experiment 2, old words studied twice for Experiment 3, and very-low frequency words for Experiment 4. The decision-making variable, *x*(*t*), dashed line, was simulated according to Equation 1, and the motor preparation variable, *y*(*t*), plain line, was simulated according to Equation 3. Variations in the amount of smoothing at the motor preparation level between tasks are due to variations in the leak parameter λ. These variations are not surprising because parameter λ is under strategic control. GCDF = gated cascade diffusion model with a filtering mechanism at the motor preparation level; a.u = arbitrary units. See the online article for the color version of this figure.

Best fitting parameters for the full models are shown in Table 1 (main parameters) and Table 2 (between-trial variability parameters). Best fitting parameters for the raw models are shown in Table C1. As predicted, both raw and full GCDF capture the EMG data with a low level of leakage, indicating strong filtering of the evidence accumulation variable during motor preparation (model trajectories for decision making and motor preparation variables computed from best fitting parameters averaged across subjects are illustrated in Figure 4A). Note that the amount of between-trial variability in the best fitting full model components was higher for GCD compared to GCDF, especially for residual latencies (parameters *sTe* and *sTr*).

**Figure 5:**
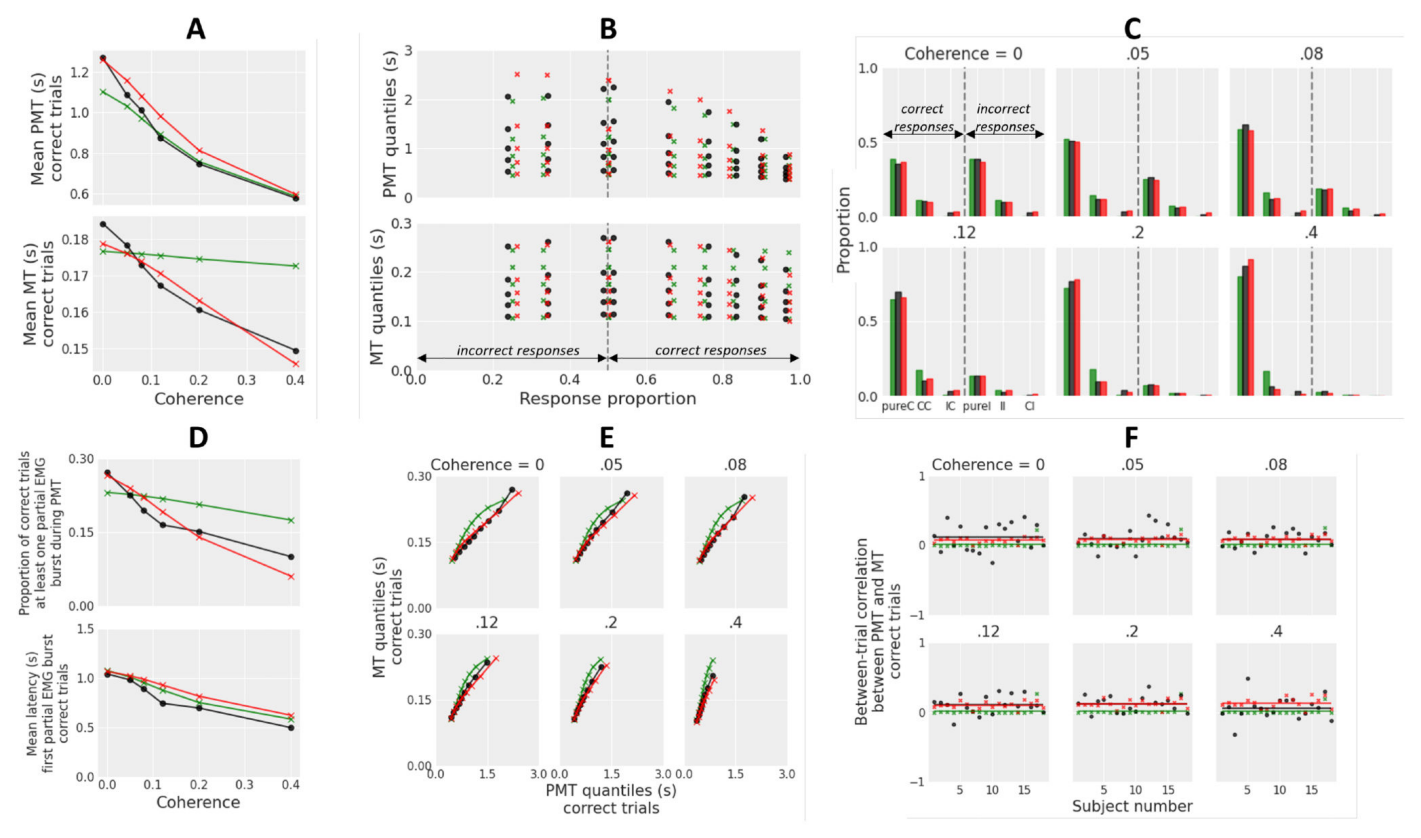
Data From a Random Dot Motion Task With Varying Levels of Motion Coherence (Black) Plotted Against Predictions From the Full GCDF (Red) and the Full GCD (Green) *Note*. Model predictions are computed from best fitting parameters, using 100,000 simulated trials per condition. The six panels (A)–(F) display different aspects of the data. (A) Mean PMT (y-axis, upper plot) and mean MT (y-axis, lower plot) in correct trials as a function of motion coherence (x-axis) averaged across subjects. (B) Quantile probability functions averaged across subjects for each motion coherence condition, constructed by plotting PMT quantiles (.1, .3, .5, .7, .9; y-axis, upper plot) and MT quantiles (.1, .3, .5, .7, .9; y-axis, lower plot) of the distributions of correct and incorrect responses against the corresponding response type proportion (x-axis). The five PMT and MT quantiles for incorrect responses in a given condition are displayed if each subject made at least 10 errors (coherence conditions 0, .05, and .08 fulfilled this requirement). (C) Proportion of each of the six trial types described in the introduction section of this experiment (pureC = pure-correct trial, CC = correct-correct trial, IC = incorrect-correct trial, pureI = pure-incorrect trial, II = incorrect-incorrect trial, CI = correct-incorrect trial) for each condition averaged across subjects. (D) Proportion of correct trials featuring at least one partial EMG burst during PMT (y-axis, upper plot) and mean latency of the first partial burst (y-axis, lower plot) as a function of motion coherence (x-axis) averaged across subjects. (E) MT quantiles (y-axis) plotted against PMT quantiles (x-axis) from correct trials for each condition averaged across subjects. Quantiles are computed from nine decile points. (F) Between-trial Pearson correlation coefficient between PMT and MT in correct trials for each condition. Observed data and model predictions for each individual subject are shown as scattered dots and crosses. Observed data and model predictions averaged across subjects are shown as horizontal lines. EMG = electromyographic; GCD = gated cascade diffusion model; GCDF = gated cascade diffusion model with a filtering mechanism at the motor preparation level; MT = motor time; PMT = premotor time. See the online article for the color version of this figure.

Figure 5 displays the goodness-of-fit of the full models to several aspects of the data. GCDF predictions are displayed in red, GCD predictions in green, and the data in black. Figure 5A shows observed versus predicted mean PMT (upper plot) and mean MT (lower plot) in correct trials averaged across subjects. Figure 5B displays observed versus predicted quantile probability functions for both PMT (upper plot) and MT (lower plot) distributions averaged across subjects. Quantile probability functions are constructed by plotting PMT or MT quantiles (y-axis) of the distributions of correct and incorrect responses for each condition against the corresponding response type proportion (x-axis). Five quantiles (.1, .3, .5, .7, .9) were chosen to provide a summary of the shape of PMT and MT distributions. If PMT and MT are uniformly distributed, the temporal separation between adjacent quantiles should be constant. If PMT and MT both exhibit a right-skewed shape, as evidenced by Servant et al. (2021) and visible in Figure 5B, the temporal separation between .7 and .9 quantiles should be larger than the separation between .5 and .7 quantiles, the separation between .5 and .7 quantiles should be larger than the separation between .3 and .5 quantiles, and so forth. Quantile probability functions thus represent a concise way to examine the shape of PMT and MT distributions for correct and incorrect responses, and how this shape varies across conditions (for a thorough treatment of quantile probability functions, see Ratcliff and Smith, 2004). Note that the five PMT and MT quantiles for incorrect responses in a given condition are displayed if each subject made at least 10 errors in that condition. Figure 5C shows the observed versus predicted proportion for each of the six trial types (pureC, CC, IC, pureI, II, CI) averaged across subjects. Figure 5D shows the observed versus predicted proportion of correct trials featuring at least one partial EMG burst during PMT (upper plot), and the mean latency of the first partial EMG burst averaged across subjects (lower plot). Figure 5E displays the observed versus predicted PMT quantile-MT quantile plot (computed from nine decile points) from correct trials averaged across subjects. Finally, Figure 5F shows the observed versus predicted between-trial Pearson correlation coefficient between PMT and MT in correct trials for each subject (scattered dots and crosses), as well as the correlation averaged across subjects (horizontal lines). The data shown in the lower plot of Figure 5D (mean latency of the first partial EMG burst in correct trials) and Figure 5F (between-trial Pearson correlation between PMT and MT) were not used to constrain parameter estimation and serve as a generalization test of the models.

Overall, the full GCDF provides a good account of the data, though two minor discrepancies are apparent. First, the model overestimates the .9 quantile of PMT distributions as motion coherence decreases, especially for incorrect trials. Second, the predicted between-trial correlation between PMT and MT for each individual subject shows less dispersion compared to observed data, but this phenomenon is likely due to noise in EMG onset detection. In addition, GCDF slightly overestimates the correlation for the highest motion coherence level. As discussed previously (see general introduction and Appendix D), the model predicts a positive correlation when a high drift rate is combined with a low leakage level, especially if between-trial variability in drift rate is incorporated.

In its raw form, GCD grossly overestimates the proportions of trials containing at least one partial EMG burst during PMT, replicating the failure of the model highlighted in the general introduction section. Since this failure was apparent in each of the four experiments presented in this article, the raw GCD will no longer be discussed. The full GCD provides a better account of the six trial types (pureC, CC, IC, pureI, II, CI), though the model overestimates the proportion of CC trials as motion coherence increases. The better performance of the full GCD comes from a much smaller response threshold *r*, but this modulation has several negative consequences. Most importantly, the predicted mean MT essentially corresponds to parameter *Tr*, which is implausible from a physiologial perspective, and the predicted variability in MT is mostly driven by parameter *sTr*. The model thus strongly underestimates the effect of motion coherence on mean MT and fails to account for the right-skewed distribution of MTs (observe the constant temporal separation between adjacent MT quantiles predicted by the model in Figure 5B, diagnostic of a uniform distribution).

## Experiment 2: Numerical Cognition

Many tasks in numeracy research involve a decision between two responses based on the magnitude of some nonsymbolic stimulus. For example, subjects have to determine which of two arrays that are spatially separated feature the larger amount of dots, or whether an array of dots contains more blue or yellow dots. Here we use another common task in numeracy research in which subjects have to determine whether the number of dots (range: 31-70) randomly scattered in a 10×10 virtual array is greater or less than a criterion quantity (50). Performance is slower and less accurate when the difference between the number of dots and the criterion is small (e.g., 45 or 55 dots) compared to when it is large (e.g., 31 or 70 dots). Ratcliff and colleagues have demonstrated that the diffusion model captures RT distributions for correct and incorrect responses and accuracy data in this task with a variation of drift rate across numerosity conditions (e.g., Ratcliff and Childers, 2015; Ratcliff et al., 2010). Ratcliff and McKoon (2018) further showed that the modulation of drift rate could arise from an approximate number representation in which numerosities are represented as Gaussian distributions, with the mean and standard deviation of these distributions increasing linearly with numerosity (Dehaene, 2003). In this framework, the drift rate corresponds to the difference between the number of dots and the criterion, scaled by a free parameter (to account for interindividual differences in discrimination performance). Consequently, both GCDF and GCD predict an increase in mean MT as the number of dots approaches the criterion, resulting in an inverted U-shaped function of numerosity (with a peak around 50). Methodological details regarding the experiment and the modeling of the data are provided in Appendix E.

### Results

#### Behavior and EMG

The data from 24 subjects were grouped into eight conditions (31-35 dots; 36-40; 41-45; 46-50; 51-55; 56-60; 61-65; 66-70), represented by the mean number of dots of each bin (33, 38, 43, 48, 53, 58, 63, 68). They were analyzed by means of quadratic contrasts (two-sided) with numerosity as within-subjects factor and specific error terms (as recommended for within-subjects designs; e.g., Boik, 1981). Anticipations (RTs < 150 ms; 0%) and trials in which participants failed to respond before the 4 s deadline (0.12%) were discarded from analyses.

**Figure 6:**
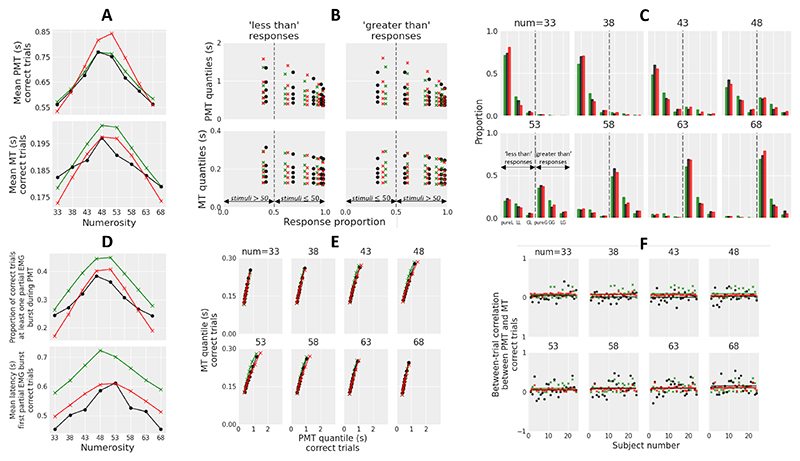
Data From a Numerosity Judgment Task (Black) and Predictions From the Full GCDF (Red) and the Full GCD (Green) *Note*. The structure of each panel is similar to that of Figure 5. Model predictions are computed from best fitting parameters, using 100,000 simulated trials per condition. Quantile probability functions, shown in Panel B, incorporate PMT and MT quantiles of incorrect responses in a given condition if each subject made at least 10 errors (numerosity conditions 48 and 53 fulfilled this requirement). Panel C shows the proportion of each of the six trial types (pureL = pure-less trial, LL = less-less trial, GL = greater-less trial, pureG = pure-greater trial, GG = greater-greater trial, LG = less-greater trial) for each numerosity condition averaged across subjects. MT = motor time; PMT = premotor time; EMG = electromyographic; GCD = gated cascade diffusion model; GCDF = gated cascade diffusion model with a filtering mechanism at the motor preparation level. See the online article for the color version of this figure.

Accuracy data exhibited a U-shaped function of numerosity, *t*(23) = 31.36, *p* < .001, reflecting the increased proportion of errors as numerosity approaches the criterion. Consistent with model predictions, mean RT, mean PMT, and mean MT showed an inverted U-shaped function of numerosity, Figure 6A; mean RT: *t*(23) = -10.78, *p* < .001; mean PMT: *t*(23) = -10.75, *p* < .001; mean MT: *t*(23) = -5.37, *p* < .001. Both the proportion of correct trials containing at least one partial EMG burst and the mean latency of the first partial burst also exhibited an inverted U-shaped function of numerosity, *t*(23) = -9.61, *p* < .001 and *t*(23) = -6.95, *p* < .001, respectively (Figure 6D). For each condition, PMT quantile-MT quantile plots from correct trials had an approximately linear shape, and the between-trial Pearson correlation coefficient between PMT and MT was positive and close to zero on average (with a slight initial reduction followed by a more pronounced increase as numerosity increases; Figure 6F). Overall, EMG results as a function of task difficulty are similar to those observed in the random dot motion task (Servant et al., 2021).

#### Model Fits

The fit procedure was identical to that used in Experiment 1, except that we treated the starting point *x*_0_ of the decision-making process as a free parameter. We modeled "less than 50" and "greater than 50" responses (with negative evidence favoring the "less" response, and positive evidence favoring the "greater" response). The six trial types considered in the fit procedure were pure-less trial (pureL: "less" response, no partial EMG burst during PMT), less-less trial (LL: "less" response, at least one partial EMG burst during PMT, first partial burst located in the "less" EMG channel), greater-less trial (GL: "less" response, at least one partial EMG burst during PMT, first partial burst located in the "greater" EMG channel), and so forth for "greater" responses (pure-greater trial pureG, greater-greater trial GG, less-greater trial LG).

Similar to Experiment 1, GCDF was associated with lower AIC and BIC statistics compared to GCD for each of the 24 subjects and for both raw and full model variants (Figure 3B). The difference in AIC and BIC between raw and full models was much smaller for GCDF compared to GCD, and the raw GCDF was associated with a lower AIC (BIC) compared to the full GCD for 21 (22) subjects. This analysis provides strong evidence for the superiority of GCDF.

Best fitting parameters for the full models are shown in Table 1 (main parameters) and Table 2 (between-trial variability parameters). Best fitting parameters for the raw models are shown in Table C1. Although the best fitting leakage (λ) value from GCDF was larger than that observed in Experiment 1, this value still implies substantial smoothing of the evidence accumulation variable during motor preparation, though with a reduced filter-related delay (for an illustration of model trajectories, see Figure 4B). The amount of between-trial variability in the best fitting full model components was higher for GCD compared to GCDF, especially for residual latencies (parameters *sTe* and *sTr*).

Figure 6 displays the goodness-of-fit of the full models to data. Compared to Experiment 1, the full GCD provides a better account of the task difficulty effect on mean MT, thanks to a larger response threshold. However, the model still fails to provide a good fit to MT quantiles because the contribution of residual motor latencies to predicted MTs remains substantial. In addition, the full GCD systematically overestimates the rate of correct LL and GG trials and the mean latency of the first partial EMG burst. The full GCDF captures most trends of the data. The only apparent misfit is an overestimation of the right skew of PMT distributions for the most difficult conditions. Note that the model provides a reasonable account of between-trial Pearson correlation coefficients between PMT and MT across numerosity conditions and does so with a complex combination of three ingredients: (a) a moderate and constant level of leakage across conditions, (b) drift rates that follow a U-shaped function of numerosity, and (c) a moderate amount of variability in drift rate that slightly increases as numerosity increases (from 0.0896 to 0.0925, computed from best fitting parameters using Equation E2 in Appendix E).

## Experiment 3: Recognition Memory

The diffusion model was originally developed to provide a theory of memory retrieval and showed a good fit to behavioral data from different item recognition paradigms (Ratcliff, 1978). This finding has been replicated multiple times since (e.g., Ratcliff et al., 2004, 2010). Here, we perform an EMG analysis of response-relevant muscles in a standard study-test task. During the study phase, participants had to memorize a list of words, each word being presented individually at a pace of 1 s. During the test phase, studied words were intermixed with nonstudied words, and participants had to decide whether each word was old or new by pressing a left or right button. In this task, the drift rate represents the meeting point between decision making and memory systems: it is equal to the amount of match between the test item and the memory trace. To modulate the drift rate, we manipulated the number of word repetitions during the study phase. Specifically, each word was studied one time, two times, or four times. The drift rate should increase as the number of repetitions (and thus memory strength) increases. Consequently, both GCD and GCDF predict a decrease in mean MT as memory strength increases.

Although early applications of the diffusion model to recognition memory data assumed a constant between-trial variability in drift rate (parameter *sv*) between old and new items, there is evidence from both memory models (e.g., Ratcliff et al., 1992; Shiffrin and Steyvers, 1997; Wixted, 2007) and diffusion model fits (e.g., Starns and Ratcliff, 2014) that the evidence entering the decision process is more variable for old than new items. One possible reason is that some old items are better learned than others (Wixted, 2007). Consequently, we let *sv* free to vary between conditions. Methodological details regarding the experiment and the modeling of the data are provided in Appendix E.

### Results

#### Behavior and EMG

**Figure 7:**
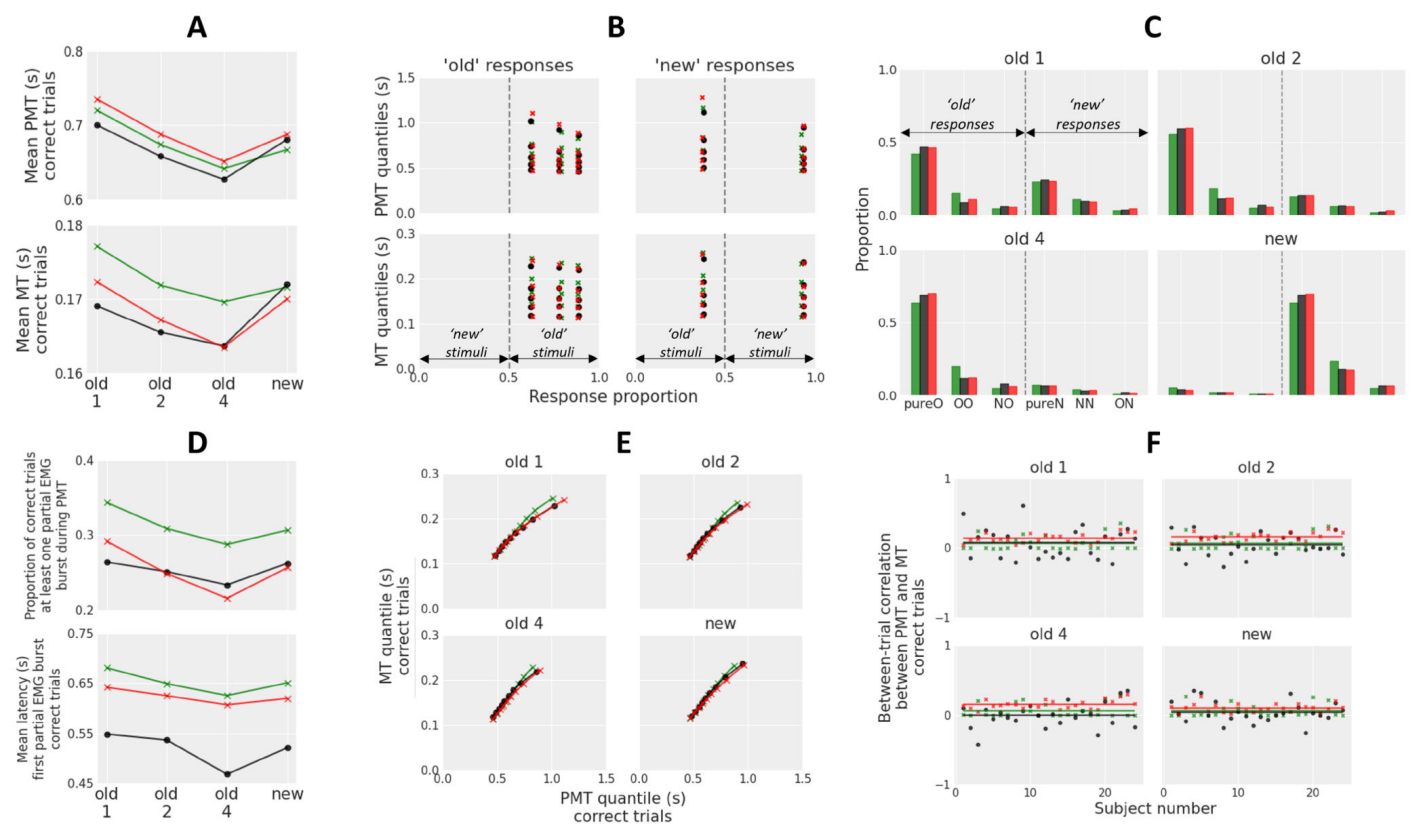
Data From a Recognition Memory Task (Black) and Predictions From the Full GCDF (Red) and the Full GCD (Green) *Note*. Conditions "old 1", "old 2", and "old 4" refer to old words studied one time, two times, and four times, respectively. The structure of each panel is similar to that of Figure 5. Model predictions are computed from best fitting parameters, using 100,000 simulated trials per condition. Quantile probability functions, shown in Panel (B), incorporate PMT and MT quantiles of incorrect responses in a given condition if each subject made at least 10 errors (condition old words studied once fulfilled this requirement). Panel C shows the proportion of each of the six trial types (pureO = pure-old trial, OO = old-old trial, NO = new-old trial, pureN = pure-new trial, NN = new-new trial, ON = old-new trial) for each condition averaged across subjects. MT = motor time; PMT = premotor time; EMG = electromyographic; GCD = gated cascade diffusion model; GCDF = gated cascade diffusion model with a filtering mechanism at the motor preparation level. See the online article for the color version of this figure.

Twenty-four subjects completed the experiment. Anticipations (RTs < 150 ms; 0.004%) and trials in which participants failed to respond before the 4 s deadline (0.068%) were discarded from analyses. Performance to old words was analyzed by means of linear contrasts (two-sided) with memory strength (words studied one time, two times, or four times) as within-subjects factor and specific error terms. Accuracy increased as memory strength increased, *t*(23) = 14.14, *p* < .001. Consistent with model predictions, mean RT, mean PMT, and mean MT decreased as memory strength increased, Figure 7A; mean RT: *t*(23) = -5.72, *p* < .001; mean PMT: *t*(23) = -5.50, *p* < .001; mean MT: *t*(23) = -3.11, *p* = .011. Note that the amplitude of the memory strength effect on mean MT (*M* = 5 ms) is smaller compared to the numerosity effect observed in Experiment 2 (*M* = 10 ms) and the motion coherence effect observed in Experiment 1 (*M* = 35 ms). The amplitude of the memory strength effect on mean PMT data (*M* = 79 ms) is also smaller compared to the numerosity effect (*M* = 198 ms) and the motion coherence effect (*M* = 692 ms). The positive correlation between the magnitude of difficulty effects on mean PMT and mean MT across tasks is consistent with the hypothesis -core to GCD and GCDF- that PMT and MT are driven by a similar evidence accumulation process.

Although both the proportion of correct trials containing at least one partial EMG burst and the mean latency of the first partial burst decreased as memory strength increased (Figure 7D), only the latter reached statistical significance, *t*(23) = -1.91, *p* = .069 and *t*(23) = -3.02, *p* = .006, respectively. For each condition, PMT quantile-MT quantile plots from correct trials exhibited a slight curvilinearity (Figure 7E). The between-trial Pearson correlation coefficient between PMT and MT was close to zero on average and slightly decreased as memory strength increased (words studied one time: *r* = .07; words studied two times: *r* = .04; words studied four times: *r* = 0; Figure 7F).

To compare performance between old and new items, we averaged the performance to old items across memory strength levels and ran two-sided paired sample *t* tests. The only significant difference concerned accuracy data. The proportion of correct responses was higher for new than old items, *t*(23) = 5.56, *p* < .001.

#### Model Fits

The fit procedure was identical to that used in the previous experiments. We modeled "new" and "old" responses (with negative evidence favoring the "new" response and positive evidence favoring the "old" response). The six trial types considered in the fit procedure were pure-old trial (pureO: "old" response, no partial EMG burst during PMT), old-old trial (OO: "old" response, at least one partial EMG burst during PMT, first partial burst located in the "old" EMG channel), new-old trial (NO: "old" response, at least one at least one partial EMG burst during PMT, first partial burst located in the "new" EMG channel), and so forth for "new" responses (pure-new trial pureN, new-new trial NN, old-new trial ON).

The raw GCDF was associated with lower AIC and BIC statistics compared to the raw GCD for each of the 24 subjects, and the full GCDF was associated with lower AIC and BIC statistics compared to the full GCD for 23 subjects (Figure 3C). The difference in AIC and BIC between raw and full model variants was smaller for GCDF compared to GCD, and the raw GCDF was associated with a lower AIC (BIC) compared to the full GCD for 16 (17) subjects. The pattern of model selection results is thus similar to that observed in the previous experiments and provides strong evidence for GCDF.

Best fitting parameters for the full models are shown in Table 1 (main parameters) and Table 2 (between-trial variability parameters). Best fitting parameters for the raw models are shown in Table C1. GCDF captures the data with a moderate amount of leakage (*λ*), though the best fitting value for the full model is a bit larger compared to Experiment 2 (implying reduced smoothing and filter-related delays; for an illustration of model trajectories, see Figure 4C). The amount of between-trial variability in the best fitting full model components was generally higher for GCD compared to GCDF. Note that between-trial variability in drift rate (*sv*) was larger for old than new words, consistent with previous work. It also decreased as memory strength increased, suggesting that evidence variability decreases as function of learning.

Figure 7 displays the goodness-of-fit of the full models to data. The full GCD provides a poor account of MT distributions due to the large contribution of residual motor latencies to predicted MTs. In addition, the full GCD systematically overestimates the proportion of correct OO and NN trials and the mean latency of the first partial EMG burst. The full GCDF provides a good fit to data used to constrain parameter estimation (though it slightly overestimates the .9 quantile of PMT distributions for old responses as the number of word presentations decreases) but shows a relatively poor generalization performance. Although the model predicts the effect of memory strength on the mean latency of the first partial EMG burst, it systematically overestimates this latency by about 100 ms. In addition, the model overestimates the between-trial correlation between PMT and MT, especially for old words studied two and four times.

## Experiment 4: Lexical Knowledge

The ability to recognize words is essential for reading, and the lexical decision task has been widely used to study this process. In this task, subjects have to decide whether strings of letters are words or nonwords. A standard finding is that high-frequency words are recognized faster and more accurately compared to low-frequency words. Ratcliff et al. (2004) showed that the diffusion model provides a good account of performance in this task, with a decrease of drift rate as word frequency decreases. Consequently, both GCD and GCDF predict an increase in mean MT as word frequency decreases.

Later modeling work suggests that word frequency modulates other parameters of the diffusion model. Both Donkin et al. (2009) and Gomez and Perea (2014) showed a variation of mean nondecision time across word frequency levels, suggesting that frequency modulates lexical access processes (that determine how much evidence the stimulus provides for each response alternative). Tillman et al. (2017) recently showed evidence for a larger between-trial variability in drift rate for words than nonwords and for high frequency compared to low frequency words, a pattern predicted by a model of lexical retrieval (Wagenmakers et al., 2004). Consequently, the mean residual latency added to predicted PMT (*Te*), drift rate (*v*), and between-trial variability in drift rate (*sv*) parameters were free to vary across word frequency conditions in our modeling of the data. Methodological details regarding the experiment and the modeling of the data are provided in Appendix E.

### Results

#### Behavior and EMG

Twenty-four subjects completed the experiment. Anticipations (RTs < 150 ms; 0%) and trials in which participants failed to respond before the 4 s deadline (0.1%) were discarded from analyses. Performance to word stimuli was analyzed by means of linear contrasts (two-sided) with word frequency (very low, low, medium, high) as within-subjects factor and specific error terms.

**Figure 8:**
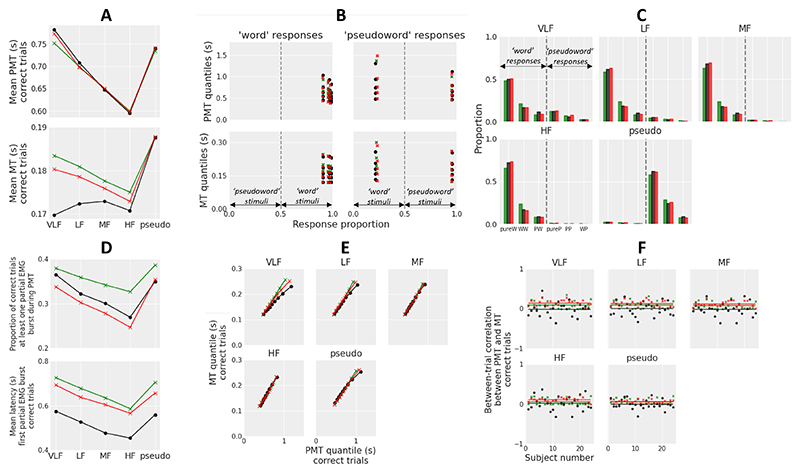
Data From a Lexical Decision Task (Black) and Predictions From the Full GCDF (Red) and the Full GCD (Green) *Note*. The structure of each panel is similar to that of Figure 5. Model predictions are computed from best fitting parameters, using 100,000 simulated trials per condition. Quantile probability functions, shown in panel (B), incorporate PMT and MT quantiles of incorrect responses in a given condition if each subject made at least 10 errors (condition very-low frequency words fulfilled this requirement). Panel (C) shows the proportion of each of the six trial types (pureW = pure-word trial, WW = word-word trial, PW = pseudoword-word trial, pureP = pure-pseudoword trial, PP = pseudoword-pseudoword trial, WP = word-pseudoword trial) for each condition averaged across subjects. VLF = very-low frequency words; LF = low frequency words; MF = medium frequency words; HF = high frequency words; pseudo = pseudowords; PMT = premotor time; MT = motor time; EMG = electromyographic; GCD = gated cascade diffusion model; GCDF = gated cascade diffusion model with a filtering mechanism at the motor preparation level. See the online article for the color version of this figure.

Accuracy decreased, *t*(23) = 12.54, *p* < .001, and mean RT increased, *t*(23) = -8.36, *p* < .001, as word frequency decreased, reflecting the classic word frequency effect. Although mean PMT decreased as word frequency decreased, *t*(23) = -8.70, *p* < .001, mean MT exhibited an unexpected inverted U-shape function of word frequency (Figure 8A). Accordingly, the planned linear contrast was not significant, *t*(23) = 0.53, *p* = .60, while a post hoc quadratic contrast reached significance, *t*(23) = -2.24, *p* = .04. This inverted U-shape pattern is unlikely due to a statistical power or an EMG signal quality issue because (a) the sample size was identical to Experiment 3, (b) the amplitude of the word frequency effect on mean RT (*M* = 187 ms) was larger than the amplitude of the memory strength effect on mean RT (*M* = 79 ms), and (c) EMG signal quality was approximately similar between Experiments 3 and 4, as revealed by a comparable percentage of rejected trials on average.

Both the proportion of correct trials containing at least one partial EMG burst during PMT and the mean latency of the first partial burst decreased as word frequency increased, *t*(23) = -6.13, *p* < .001 and *t*(23) = -6.39, *p* < .001, respectively (Figure 8D). For each condition, PMT quantile-MT quantile plots from correct trials exhibited an approximately linear shape (Figure 8E), and the between-trial Pearson correlation coefficient between PMT and MT was remarkably close to zero on average, with no apparent trend across conditions (Figure 8F).

To compare the performance between word and pseudoword stimuli, we averaged the performance to word stimuli across frequency levels and ran two-sided paired sample *t* tests. The proportion of correct responses was higher for pseudowords than words, *t*(23) = -4.24, *p* < .001. Mean RT, mean PMT, and mean MT were slower for pseudowords than words, *t*(23) = -4.40, *p* < .001, *t*(23) = -3.35, *p* = .003, and *t*(23) = -2.49, *p* = .02, respectively. Finally, there was a trend for a smaller proportion of correct trials containing at least one partial EMG burst for words than pseudowords, *t*(23) = -2.05, *p* = .052, and the mean latency of the first partial burst was faster for words, *t*(23) = -4.21, *p* < .001.

#### Model Fits

The fit procedure was identical to that used in the previous experiments. We modeled "pseudoword" and "word" responses (with negative evidence favoring "pseudoword" responses and positive evidence favoring "word" responses). The six trial types considered in the fit procedure were pure-word trial (pureW: "word" response, no partial EMG burst during PMT), word-word trial (WW: "word" response, at least one partial EMG burst during PMT, first partial burst located in the "word" EMG channel), pseudoword-word trial (PW: "word" response, at least one partial EMG burst during PMT, first partial burst located in the "pseudoword" EMG channel), and so forth for "pseudoword" responses (pure-pseudoword trial pureP, pseudoword-pseudoword trial PP, word-pseudoword trial WP).

GCDF was associated with lower AIC and BIC statistics compared to GCD for each of the 24 subjects and for both raw and full model variants (Figure 3D). The difference in AIC and BIC between raw and full model variants was smaller for GCDF compared to GCD, and the raw GCDF was associated with a lower AIC (BIC) compared to the full GCD for 14 (15) subjects. The pattern of model selection results is thus similar to that observed in the previous experiments and provides strong evidence for GCDF.

Best fitting parameters for the full models are shown in Table 1 (main parameters) and Table 2 (between-trial variability parameters). Best fitting parameters for the raw models are shown in Table C1. GCDF captures the data with a higher level of leakage *λ* compared to the previous experiments (implying reduced smoothing and filter-related delays; for an illustration of model trajectories, see Figure 4D). Consistent with previous work (Donkin et al., 2009; Gomez and Perea, 2014), GCDF and GCD both predict an increase in the mean residual latency parameter *Te* added to predicted PMT as word frequency decreases. Although evidence variability (parameter *sv*) was generally larger for words than pseudowords, consistent with previous work (Tillman et al., 2017; Wagenmakers et al., 2004), it increased as word frequency decreased. The latter pattern is opposite to that found by Tillman et al. (2017) using traditional diffusion model fits. The number of words for which people do not know the definition may increase as word frequency decreases, inflating evidence variability. More generally, the amount of between-trial variability in the best fitting full model components was generally higher for GCD compared to GCDF, consistent with model fits obtained in the previous experiments.

Figure 8 displays the goodness-of-fit of the full models to data. As expected, both GCDF and GCD predict an increase in predicted mean MT as word frequency decreases, and fail to capture the observed inverted U-shaped pattern. GCD systematically underestimates the .9 quantile of PMT distributions for correct responses and overestimates the proportion of correct WW and PP trials. GCDF provides a better account of PMT distributions and the six trial types (pureW, WW, PW, pureP, PP, WP). Both models overestimate the mean latency of the first partial EMG burst and the between-trial correlation between PMT and MT.

## Comparison Between the Neural Drive to Muscle Fibers Predicted by GCDF and Full-Wave Rectified EMG Signals

As a final evaluation of GCDF, we compared the predicted neural drive to muscle fibers, variables *z*_*L*_(*t*) or *z*_*R*_(*t*) (Equation 9), with full-wave rectified EMG signals. As mentioned in the general introduction, the full-wave rectified EMG signal can be interpreted as a noisy approximation of the neural drive to the area of muscle over which the electrodes are placed, and should thus scale with the predicted neural drive computed from best fitting parameters. We restricted this analysis to EMG data from Experiments 1 and 2, in which the effects of experimental manipulations on MT were the largest and well accounted for by the model.

**Figure 9:**
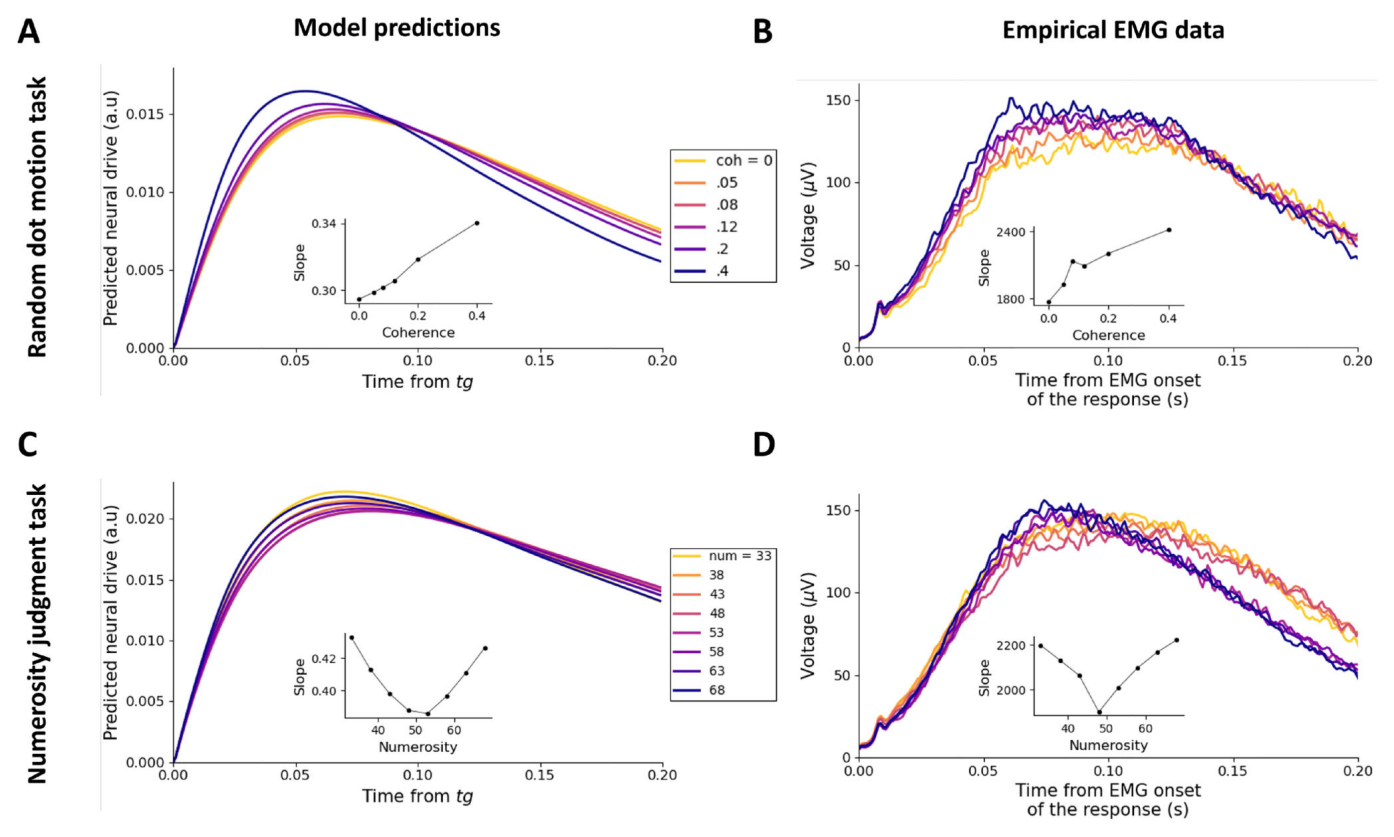
Neural drive predicted by GCDF in correct trials and corresponding full-wave rectified EMG bursts for each condition of Experiment 1 and Experiment 2 *Note*. Panels (A) and (B) show GCDF predictions and EMG data, respectively, in the random dot motion task. Panels (C) and (D) show GCDF predictions and EMG data, respectively, in the numerosity judgment task. Insets represent the rising slope of signals in each condition, estimated by linear regression in the 0–50 ms window. See text for additional details. EMG = electromyographic; a.u = arbitrary units; GCDF = gated cascade diffusion model with a filtering mechanism at the motor preparation level. See the online article for the color version of this figure.

Figure 9A and 9C show the predicted neural drive to muscle fibers associated with the correct response, averaged across correct trials and subjects for the random dot motion task and the numerosity judgment task, respectively. Model trajectories are time-locked on *t*_*g*_, latency at which *z*_*L*_(*t*) or *z*_*R*_(*t*) becomes positive for the last time before reaching the response threshold (see Figure 1). For each task, subject, and condition, 1,000 correct trials were simulated using the best fitting parameters from the full GCDF. In each simulated trial, the neural drive was assumed to decay at an arbitrary linear rate of -0.15 units/s after hitting the response threshold. Trajectories were then time locked on *t*_*g*_ and averaged. For each task, the rising slope of the predicted neural drive decreases as difficulty increases, reflecting the dynamics of the underlying motor preparation signal.

Figure 9B and 9D show full-wave rectified EMG signals from muscles associated with the correct response for each condition of the random dot motion task and the numerosity judgment task, respectively. Signals are time-locked to the EMG onset of the response, and are averaged across correct trials and subjects. Consistent with model predictions, the rising slope of EMG signals decreases as difficulty increases. A linear contrast computed on the rising slope (estimated by linear regression in the 0-50 ms window) in the random dot motion task was highly significant, *t*(27) = 4.89, *p* < .001, and so was the quadratic contrast in the numerosity judgment task, *t*(23) = 3.66, *p* < .001. This analysis provides additional evidence for GCDF.

## General Discussion

To our knowledge, this work represents the first attempt to jointly model decision making, motor preparation, and motor execution processes in choice RT tasks. The proposed GCDF assumes a continuous flow of the evidence accumulation decision variable to agonist muscles. The model further incorporates a smoothing mechanism at the motor preparation level and a gate that regulates the flow of information from motor preparation to muscle fibers. This architecture offers substantial flexibility to motor control by allowing for real-time adjustments of motor commands based on incoming evidence, while simultaneously shielding the system against unwanted behaviors and preventing unnecessary muscular activity. The smoothing mechanism at the motor preparation level may also reflect an attempt to recover the decision variable from noise that can corrupt it during the transmission process and can be seen as an approximation of a Kalman-Bucy filter.

We tested GCDF against behavioral and EMG data from four choice tasks that span a variety of domains in cognitive sciences, namely motion perception (Experiment 1), numerical cognition (Experiment 2), recognition memory (Experiment 3), and lexical knowledge (Experiment 4). Each task featured a manipulation of choice difficulty to bring additional constraints to the model. GCDF was evaluated in its ability to capture (a) the shape of PMT and MT distributions for correct and incorrect responses; (b) the proportion of six trial types defined by the combination of response type, presence versus absence of at least one partial EMG burst during PMT, and EMG channel location of the first partial burst; (c) the mean latency of the first partial EMG burst in correct trials; (d) the relationship between the shape of PMT and MT distributions in correct trials; (e) the between-trial Pearson correlation coefficient between PMT and MT in correct trials; (f) the neural drive to muscle fibers; and (g) the variation of all of the above aspects of the data across difficulty conditions. Overall, GCDF provided a good fit to data used to constrain parameter estimation (a, b, and d). The only apparent discrepancy between data and model predictions was an overestimation of the .9 quantile of PMT distributions for the most difficult experimental conditions. One way to solve this issue would be to incorporate an urgency signal into the model (Cisek et al., 2009; Ditterich, 2006; Evans, Hawkins, and Brown, 2020; Hawkins et al., 2015; Trueblood et al., 2021). Urgency can take the form of temporally collapsing boundaries or a time-increasing gain applied to the incoming evidence. Both mechanisms reduce the skew of predicted RT distributions (Hawkins et al., 2015), offering a potential solution to the observed GCDF misfit. Although urgency signals remain controversial when considering behavioral data alone (Evans, Hawkins, and Brown, 2020; Glickman and Usher, 2019; Glickman et al., 2022; Hawkins et al., 2015; Ratcliff et al., 2016; Trueblood et al., 2021), neurophysiological studies have provided evidence for them at the motor preparation level in both monkeys and humans (Churchland et al., 2008; Hanks et al., 2014; Murphy et al., 2016), even when subjects are not under speed pressure (Kelly et al., 2021). Interestingly, urgency signals are not observed at the decision-making level (Kelly et al., 2021; Steinemann et al., 2018), further emphasizing the functional dissociation between decision making and motor preparation. Fitting GCDF variants that incorporate urgency mechanisms is beyond the scope of the present work and should be conducted in tandem with an electrophysiological investigation of motor preparation.

Although GCDF showed a good fit to data used to constrain parameter estimation, it provided a mixed predictive account of the remaining data. The model captured the mean latency of the first partial EMG burst in Experiments 1 and 2 but systematically overestimated this latency by about 100 ms in Experiments 3 and 4. We cannot exclude the possibility that a set of parameters could have better captured the partial burst latency data had we considered these data in parameter estimation. Alternatively, variations in EMG signal quality across experiments may have contributed to this pattern of results, as the percentage of rejected trials was larger on average in Experiments 3 (12.41%) and 4 (10.4%) compared to Experiments 1 (7.5%) and 2 (2.7%). Therefore, the data from Experiments 3 and 4 might incorporate a larger amount of artifactual partial EMG bursts.

Besides the mean latency of partial EMG bursts, GCDF provided a mixed predictive account of the between-trial correlation between PMT and MT. In general, model predictions showed more dispersion compared to observed data at the individual level, but this phenomenon is likely explained by noise in EMG onset detection. However, the model systematically overestimated the correlation averaged across subjects in Experiments 3 and 4, and the easiest condition of Experiment 1. One may argue that this discrepancy between data and model predictions speaks against the model architecture, as the filtering mechanism at the motor preparation level flattens out random fluctuations of the evidence accumulation signal and increases the predicted correlation between PMT and MT at the single-trial level. Once again, we cannot exclude the possibility that a set of parameters could have better captured the between-trial correlation between PMT and MT had we considered these data in parameter estimation. Alternatively, it is important to remember that the filtering mechanism (regulated by the leak parameter) interacts in complex ways with drift rate and between-trial variability in drift rate (see Appendix D). This interaction is problematic because GCDF variants used in Experiments 3 and 4 do not incorporate representational assumptions that specify how drift rate distributions arise from the stimuli. In addition, although the linear relationship between motion coherence and drift rate provided a good fit to data from Experiment 1 (see also Palmer et al., 2005; Ratcliff and McKoon, 2008), a more complex representational assumption has recently been proposed for the random dot motion task (Smith and Lilburn, 2020). Consequently, discrepancies between data and model predictions may stem from a misspecification of drift rate distributions.

More generally, these findings highlight the need of considering predecisional processing stages when modeling motor phenomena. This need is further highlighted by the lexical decision data from Experiment 4. The word frequency effect has been successfully modeled by assuming that word frequency modulates drift rate (Ratcliff et al., 2004), mean nondecision time (Donkin et al., 2009; Gomez and Perea, 2014), and between-trial variability in drift rate (Tillman et al., 2017). Within the framework of GCDF, the decrease of drift rate as word frequency decreases should increase the predicted mean MT. Contrary to this prediction, we found an inverted U-shape relationship between mean MT and word frequency. Specifically, mean MT showed an initial increase from high frequency to medium frequency words, followed by a decrease for low and very-low frequency words. At first glance, this result speaks against the architecture of GCDF. However, the model does not incorporate assumptions regarding how the drift rate is computed in this task, so the origin of the problem is unclear. It would be useful to connect models of lexical access (e.g., Grainger, 2018; Houghton, 2018; McClelland and Rumelhart, 1981) to GCDF to shed light on this issue.

Apart from the unexpected word frequency effect on mean MT, EMG findings were remarkably consistent across experiments, suggesting that GCDF generalizes across cognitive domains. Both mean PMT and mean MT increased as choice difficulty increased. The increase of mean MT as choice difficulty increased was caused by a decrease in the rising slope of the neural drive, as suggested by our analysis of full-wave rectified EMG signals. This important aspect of the data was nicely captured by GCDF because the neural drive predicted by the model reflects evidence-dependent dynamics of the underlying motor preparation signal. Partial bursts were also observed in the EMG data of each subject of each experiment. The proportion of correct trials containing at least one partial EMG burst during PMT and the mean latency of the first partial burst increased as choice difficulty increased. Interestingly, the proportion of correct trials in which the first partial burst was located in the same EMG channel as the response was systematically larger than the proportion of correct trials in which the first partial burst was located in the opposite EMG channel. Within the framework of GCDF, this finding is explained by the same mechanism that captures the relative proportion of correct and incorrect responses. Putting aside between-trial variability in model components, errors are produced by noise in the evidence accumulated at each time step. Although part of this noise is flattened out during motor preparation, the predicted proportion of errors is smaller than the proportion of correct responses, so long as the drift rate is not null.

### Comparisons With GCD

As predicted, GCDF captured the data with a relatively low level of leakage, indicating that the evidence accumulation variable is smoothed at the motor preparation level. This finding suggests that the smoothing mechanism adds to a GCD description of the data. Model selection statistics (AIC and BIC) further showed that the additional complexity of GCDF was justified in light of the (large) improvement in fit quality. Both AIC and BIC statistics favored GCDF over GCD for 90/90 participants (raw models) and 89/90 (full models). These findings provide decisive evidence for GCDF. Interestingly, the difference in model selection statistics between raw and full models was much larger for GCD than for GCDF, suggesting that between-trial variability in GCD components has a major impact on the fit quality of the model, contrary to GCDF. Still, the AIC (BIC) statistic favored the raw GCDF over the full GCD for 67(70)/90 subjects. Although between-trial variability in processing components is plausible, we believe that a large contribution of between-trial variability to the fit quality of a model is problematic, as there is generally no explanation of why this variability occurs or why it has the parametric form researchers assume to represent it. In this view, between-trial variability essentially corresponds to adding a random component to the model without any strong theoretical motivation for it rather than to improve the fit quality (Evans, Tillman, and Wagenmakers, 2020). Consequently, we consider our findings regarding between-trial variability as additional evidence for GCDF.

In its raw form, GCD grossly overestimated the proportion of trials containing at least one partial EMG burst, especially when the first partial burst was located in the same EMG channel as the response. The full GCD provided a better account of these proportions by using a very small response threshold (Experiment 1) or by combining high drift rates with a high between-trial variability in drift rates (Experiments 2-4; see Tables 1 and 2). However, both processing schemes resulted in predicted MTs that were too fast compared to observed MTs. The model compensated this problem by increasing the contribution of residual motor latencies to predicted MTs (parameters *Tr* and *sTr*), but this compensation had two negative consequences. First, the model was not able to capture large effects of choice difficulty on mean MT, such as those observed in Experiment 1. Second, the model was not able to capture the right-skewed shape of MT distributions because between-trial variability in residual motor latencies added to predicted MTs is uniformly distributed, an (arbitrary) assumption inherited from the diffusion decision model (Ratcliff and Rouder, 1998).

### Neurophysiological Implementation, Theoretical Limitations, and Possible Model Extensions

As detailed in the general introduction, properties of motor preparation and execution, uncovered by neurophysiological studies, are not accounted for by current RT models such as the diffusion model (Ratcliff, 1978; Ratcliff et al., 2016), the leaky competing accumulator (Usher and McClelland, 2001), the linear ballistic accumulator (Brown and Heathcote, 2008), racing diffusion models (Ratcliff et al., 2003; Tillman et al., 2020), and Poisson counter models (Ratcliff and Smith, 2004; Vickers, 1970). In this respect, we believe that the models proposed by Servant et al. (2015, 2021), Verdonck et al. (2021), and their development and integration through GCDF constitute a major theoretical advance in the field, as they offer a mechanistic explanation to the interplay between decision and motor processes.

The diffusion decision process has been the object of several neurocomputational characterizations and has been hypothesized to arise from recurrent loops within neural networks (Smith and McKenzie, 2011; Wang, 2002; Wong and Wang, 2006). Interestingly, recurrent neural networks have also been used to approximate Kalman-Bucy filters (Denève et al., 2007), paving the way for a joint characterization of decision and motor preparation processes at the neural circuit level. At the systems level, we believe that future empirical tests of GCDF would benefit from a combination of EEG and EMG recordings. Although the application of a low-pass filter during EEG signal processing precludes a precise test of the Kalman-Bucy filter hypothesis at the motor preparation level, averaged model trajectories at decision making and motor preparation levels computed from best fitting parameters could be compared to CPP and effector-selective motor preparation EEG activities, respectively. Some parameters of the model could also be constrained to match corresponding electrical signatures (Kelly et al., 2021).

Additional constraints to GCDF could also arise from a more detailed analysis of partial EMG bursts at the motor execution level. Some trials contain more than one partial burst during PMT, and these additional bursts could be considered in the modeling. In particular, the co-occurrence of two partial bursts in left and right EMG channels would suggest some degree of independence between accumulators. We note, however, that more detailed EMG analyses entail an increased sensitivity to potentially artifactual electrical activities. In addition, partial EMG bursts might be followed by a refractory period. Consequently, the analysis and modeling of trials with multiple partial bursts represent an important challenge for future work.

Beyond the basic mechanisms that drive the time course of decision making, motor preparation, and motor execution processes, we believe that future developments of GCDF would benefit from model-based investigations of more complex relationships between decision and motor processes. For example, decisions are often taken well before being expressed behaviorally. This scenario is involved when voters have to choose a candidate. It is currently outside the scope of GCDF, as the model does not specify the relationship between memory and decision/motor processes. The choice might be categorically retrieved from memory and transmitted to the motor system. Consequently, effector-selective motor preparation EEG activities and EMG signals should not be modulated by the quality of evidence. However, this hypothetical processing scheme may vary as a function of the temporal delay between decision and motor processes and foreknowledge of the stimulus-response mapping (Twomey et al., 2016).

Another scenario that deserves additional scrutiny concerns continuous movement reports. Similar to EMG findings, reaching trajectories are modulated by perceptual and cognitive factors (e.g., Buc Calderon et al., 2015; Kinder et al., 2022; Song and Nakayama, 2009; Sullivan et al., 2015). However, the application of GCDF to choice reaching tasks is not straightforward. Reaching movements engage a complex pattern of neuromuscular activity, making EMG recordings and analyses challenging. One way to reduce this complexity is to model reaching movements at the level of kinematic motor primitives, hypothetical building blocks that can be combined to construct motion (for reviews, see Flash and Hochner, 2005; Giszter, 2015; Latash, 2020). Despite their apparent continuity, reaching movements appear to be composed of discrete submovements. Friedman et al. (2013) hypothesized that an intermittent motor control process probes the state of accumulated evidence at discrete time points to determine submovements and showed good fits of this model to arm movement trajectories in a variant of the random dot motion task. The relationship between this intermittent motor control process, motor preparation, and EMG activity remains to be elucidated.

To conclude, the present EMG investigations in choice RT tasks add to a growing body of behavioral and neurophysiological evidence that suggests that the motor system can have systematic effects that are computationally related to central decision processes. These effects are important to complete the story of how our choices are reflected in our actions. The proposed GCDF offers a new framework to understand this relationship.

## Supplementary Material

Appendix

## Figures and Tables

**Table 1:** Main Parameters From the Full GCD and GCDF Models Averaged Across Subjects for Experiments 1–4

Exp	Model	*v*_1_	*v*_2_	*v*_3_	*v*_4_	*v*_5_	*k*	*dc*	*λ*	*g*	*r*	*Te*_1_	*Te*_2_	*Te*_3_	*Te*_4_	*Te*_5_	*Tr*	*x*_0_
1	GCD						1.534			0.091	0.005	0.353					0.169	
1	GCDF						1.183		21.471	0.069	0.022	0.283					0.093	
2	GCD	0.066						-0.035		0.065	0.036	0.474					0.148	0.006
2	GCDF	0.023						-0.011	57.842	0.042	0.030	0.339					0.106	0.000
3	GCD	0.207	0.518	0.739	-0.656					0.045	0.024	0.544					0.139	0.001
3	GCDF	0.138	0.288	0.399	-0.342				73.158	0.037	0.031	0.452					0.099	0.000
4	GCD	0.459	0.683	0.809	0.878	-0.571				0.046	0.036	0.589	0.571	0.549	0.512	0.581	0.133	-0.004
4	GCDF	0.277	0.393	0.460	0.517	-0.301			122.938	0.041	0.035	0.500	0.471	0.454	0.430	0.489	0.109	-0.006

*Note*. Experiment 3 (recognition memory): subscripts 1-4 for parameter *v* correspond to conditions old words studied one time, old words studied two times, old words studied four times, and new words, respectively. Experiment 4 (lexical knowledge): subscripts 1-5 for parameters *v* and *Te* correspond to conditions very-low frequency words, low frequency words, medium frequency words, high frequency words, and pseudowords, respectively. GCD = gated cascade diffusion model; GCDF = gated cascade diffusion model with a filtering mechanism at the motor preparation level.

**Table 2:** Between-trial variability parameters from the full GCD and GCDF models averaged across subjects for Experiments 1-4.

Exp	Model	*sv*_1_	*sv*_2_	*sv*_3_	*sv*_4_	*sv*_5_	*σ*_1_	*η*_0_	*sx*_0_	*sTe*	*sTr*	*s*λ
1	GCD	0.134							0.085	0.286	0.168	
1	GCDF	0.117							0.074	0.203	0.076	29.423
2	GCD						0.007	0.050	0.098	0.422	0.133	
2	GCDF						0.001	0.042	0.060	0.225	0.078	82.803
3	GCD	0.439	0.427	0.426	0.274				0.065	0.327	0.113	
3	GCDF	0.335	0.313	0.263	0.163				0.052	0.204	0.065	86.538
4	GCD	0.522	0.435	0.372	0.312	0.267			0.071	0.456	0.107	
4	GCDF	0.334	0.266	0.208	0.195	0.122			0.057	0.350	0.075	146.702

*Note*. Experiment 3 (recognition memory): parameters *sv*_1_ to *sv*_4_ correspond to between-trial variability in drift rate for conditions old words studied one time, old words studied two times, old words studied four times, and new words respectively. Experiment 4 (lexical knowledge): parameters *sv*_1_ to *sv*_5_ correspond to between-trial variability in drift rate for conditions very low frequency words, low frequency words, medium frequency words, high frequency words, and pseudowords respectively. Experiment 3 (recognition memory): subscripts 1-4 for parameter *sv* correspond to conditions old words studied one time, old words studied two times, old words studied four times, and new words, respectively. Experiment 4 (lexical knowledge): subscripts 1-5 for parameter sv correspond to conditions very-low frequency words, low frequency words, medium frequency words, high frequency words, and pseudowords, respectively. GCD = gated cascade diffusion model; GCDF = gated cascade diffusion model with a filtering mechanism at the motor preparation level.

## Footnotes

1 Full-wave rectification consists of taking the absolute value of voltages across time points (so all negative voltages become positive). This transformation requires the EMG signals to be centered around zero.

2 Technically, the neural drive refers to the electrical activity of *α*-motoneurons that innervate muscle fibers. This activity triggers an excitation-contraction coupling at the level of muscle fibers, leading to force production (Calderón et al., 2014). EMG measures the electrical excitation of muscle fibers, which is a precursor to force generation.

3 Parameters *g* and *r*, respectively, correspond to parameters *m* and *r−m* in the dual-threshold diffusion model.

4 The only apparent discrepancy concerned the between-trial correlation between PMT and MT. This correlation was slightly positive on average in the data, and there was some variability between participants, presumably due to the impact of noise on EMG onset detection. Additional simulations of GCD showed that the model could predict a small positive correlation between PMT and MT for a given drift rate level when between-trial variability in drift rate is incorporated.

5 Equation 7 is still optimal in the sense that it minimizes the mean squared prediction error $E\left[{\left(x(t)-\tilde{y}(t)\right)}^{2}\right]$, but optimality here must be understood under the assumption that parameters *v, σ*, and *ξ* are replaced by priors *v′* and *λ′*. It can be contrasted with the optimal filter applied by an ideal observer – who would use the actual values of *v, σ*, and *ξ* (see Appendix C).

6 Relaxing this constraint did not change model selection results, and had a negligible impact on the goodness-of-fit of the models.

7 If subjects made a number of errors comprised between five and 10 in a given condition, a median split was used to form two bins. It there were fewer than five errors, error PMTs and MTs for the condition were excluded from the *G*^2^ calculation.
