# Supplementary material for: The Gated Cascade Diffusion Model: An Integrated Theory of Decision Making, Motor Preparation, and Motor Execution
Source: Psychol Rev. Author manuscript; Available in PMC 2024 Aug 15. (PMC7616365; doi:10.1037/rev0000464)
Supplement: Appendix [file EMS193425-supplement-Appendix.pdf]

Appendix A
Empirical Illustrations of Trials Containing at Least One Partial EMG Burst During PMT

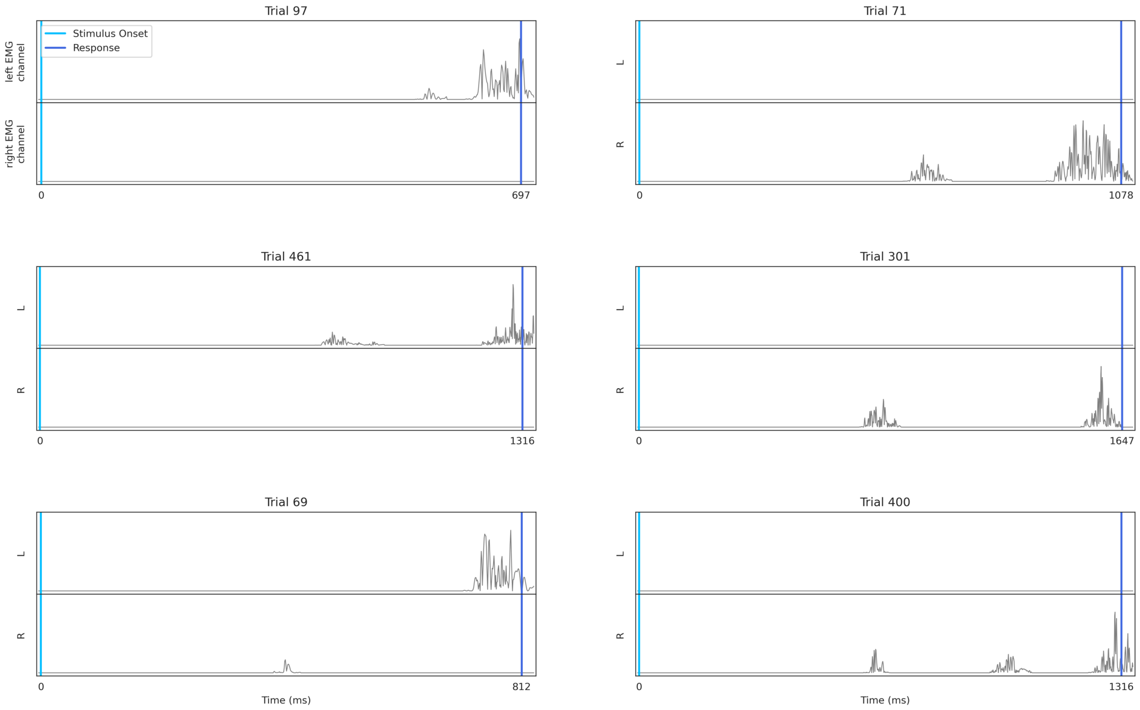

Note. Panels (A), (B), (C), and (D) show trials that contain one partial EMG burst in the same EMG channel as the response. Panel (E) shows a trial with a partial EMG burst in the opposite EMG channel as the response. Panel (F) shows a trial with two partial EMG bursts in the same EMG channel as the response. PMT = premotor time; EMG = electromyographic. See the online article for the color version of this figure.

(*Appendices continue*)

Appendix B
Mathematical details

Derivation of the Kalman–Bucy Filter at the Motor Preparation Level (Equation 6)

Definitions of the decision variable $x(t)$ and its noisy variant $\tilde{x}(t)$ received by the motor preparation system are provided in the main text by Equations 1 and 5 respectively. In order to have a tractable mathematical representation of the problem, it is better to write it down in terms of differential equations. This is already the case for $x(t)$. However, we cannot differentiate $\tilde{x}(t)$ as it stands, because we cannot differentiate a white noise. We thus introduce a new process $q(t)$, defined as $q(t) = \int_0^t \tilde{x}(s)ds$. We have:

$$dq(t) = x(t)dt + \xi dV(t), \quad q(0) = x_0, \tag{B1}$$

where $V(t)$ is a Brownian motion, independent of $W(t)$. Note that processes $q(t)$ and $\tilde{x}(t)$ contain the exact same information.

The Kalman-Bucy filtered motor preparation process $\tilde{y}(t)$ satisifes the following differential equation (Øksendal, 2003):

$$d\tilde{y}(t) = \left(v - \frac{S(t)}{\xi^2}\tilde{y}(t)\right)dt + \frac{S(t)}{\xi^2}dq(t), \quad \tilde{y}(0) = x_0, \tag{B2}$$

where $S(t) = \mathbb{E}\left[(x(t) - \tilde{y}(t))^2\right]$, and satisfies the Riccati equation:

$$\frac{dS}{dt} = -\frac{S^2}{\xi^2} + \sigma^2, \quad S(0) = 0. \tag{B3}$$

The solution to the Riccati equation is:

$$\begin{aligned} S(t) &= \frac{-\xi\sigma + \xi\sigma e^{\frac{2\sigma}{\xi}t}}{1 + e^{\frac{2\sigma}{\xi}t}} \\ &= \xi\sigma \tanh\left(\frac{\sigma}{\xi}t\right). \end{aligned} \tag{B4}$$

Substituting $S(t)$ in Equation B2, we obtain:

$$d\tilde{y}(t) = \frac{\sigma}{\xi}\tanh\left(\frac{\sigma}{\xi}t\right)(dq(t) - \tilde{y}(t)dt) + vdt, \quad \tilde{y}(0) = x_0. \tag{B5}$$

Finally, from the definition of $q(t)$, we have $dq(t) = \tilde{x}(t)dt$, which leads to:

$$d\tilde{y}(t) = \frac{\sigma}{\xi}\tanh\left(\frac{\sigma}{\xi}t\right)(\tilde{x}(t) - \tilde{y}(t))dt + vdt, \quad \tilde{y}(0) = x_0. \tag{B6}$$

Relationship Between Equation 3 and 8

In the main text, we noted that Equation 3 and Equation 8 differ with respect to the input to motor preparation. This difference can be further characterized by solving each

equation, and replacing parameter λ' by λ in Equation 8:

$$\begin{aligned} y(t) &= \lambda \int_0^t e^{\lambda(s-t)} x(s) ds \\ \tilde{y}(t) &= \lambda \int_0^t e^{\lambda(s-t)} \tilde{x}(s) ds. \end{aligned} \tag{B7}$$

Since $\tilde{x}(s)ds = dq(s) = x(s)ds + \xi dV(s)$, we obtain:

$$\begin{aligned} \tilde{y}(t) &= \lambda \int_0^t e^{\lambda(s-t)} (x(s)ds + \xi dV(s)) \\ &= \lambda \int_0^t e^{\lambda(s-t)} x(s) ds + \lambda\xi \int_0^t e^{\lambda(s-t)} dV(s) \\ &= y(t) + \lambda\xi \int_0^t e^{\lambda(s-t)} dV(s). \end{aligned} \tag{B8}$$

Using Itô isometry, we see that $\tilde{y}(t)$ is equal to $y(t)$ plus a Gaussian noise with mean 0 and variance $\varphi = \lambda\xi^2 \frac{1-e^{-2\lambda t}}{2}$. This Gaussian noise is independent from y (because V and W are independent). Note that $\varphi < \frac{\lambda\xi^2}{2}$, and $\tilde{y}(t)$ converges towards $y(t)$ as the amplitude of transmission noise ξ approaches 0.

(*Appendices continue*)

Appendix C
Fits of a GCDF Variant That Uses Variable $\tilde{y}(t)$ to Model Motor Preparation

We compared the fit performance of GCDF with a model variant that uses $\tilde{y}(t)$ computed using Equation 7. We refer to this variant as gated cascade diffusion model with a Kalman-Bucy filter at the motor preparation level (GCDK). Based on our mathematical analysis, we expected a comparable fit performance between the two models. GCDK was fit to behavioral and EMG data from the four choice tasks using a methodology identical to that described in the main text. To avoid complications arising from between-trial variability in processing components, we restricted this analysis to raw models. GCDK has one more free parameter than GCDF (the amplitude of transmission noise ξ).

Figure C1 displays model comparison statistics (AIC and BIC) for each task. The plot shows a considerable degree of overlap between GCDF and GCDK, diagnostic of a similar performance. The AIC (BIC) favors GCDK over GCDF for 7 (7) subjects for the random dot motion task, 12 (10) subjects for the numerosity judgment task, 14 (14) subjects for the recognition memory task, and 11 (10) subjects for the lexical decision task. Each of these differences fails to reach statistical significance (two-sided binomial test).

Best fitting GCDK parameters averaged across subjects are reported in Table C1. It is worth noting that the prior λ' on the ratio between diffusion noise σ and transmission noise ξ is significantly smaller than the ratio computed from an ideal observer perspective, resulting in a stronger smoothing of the corrupted decision variable $\tilde{x}$ than actually needed. This phenomenon could be interpreted as a bias toward response accuracy relative to response speed (Verdonck et al., 2021). This analysis must be taken with caution, as we suspect trade-offs between GCDK parameters (suggested by the very short residual latency Te added to predicted PMT in Experiment 1, or the very small prior λ' in Experiment 3). A comprehensive assessment of these trade-offs is beyond the scope of the present work.

Figure C1

Model Selection Statistics for Experiments 1–4, Including GCDK and Focusing on Raw Models

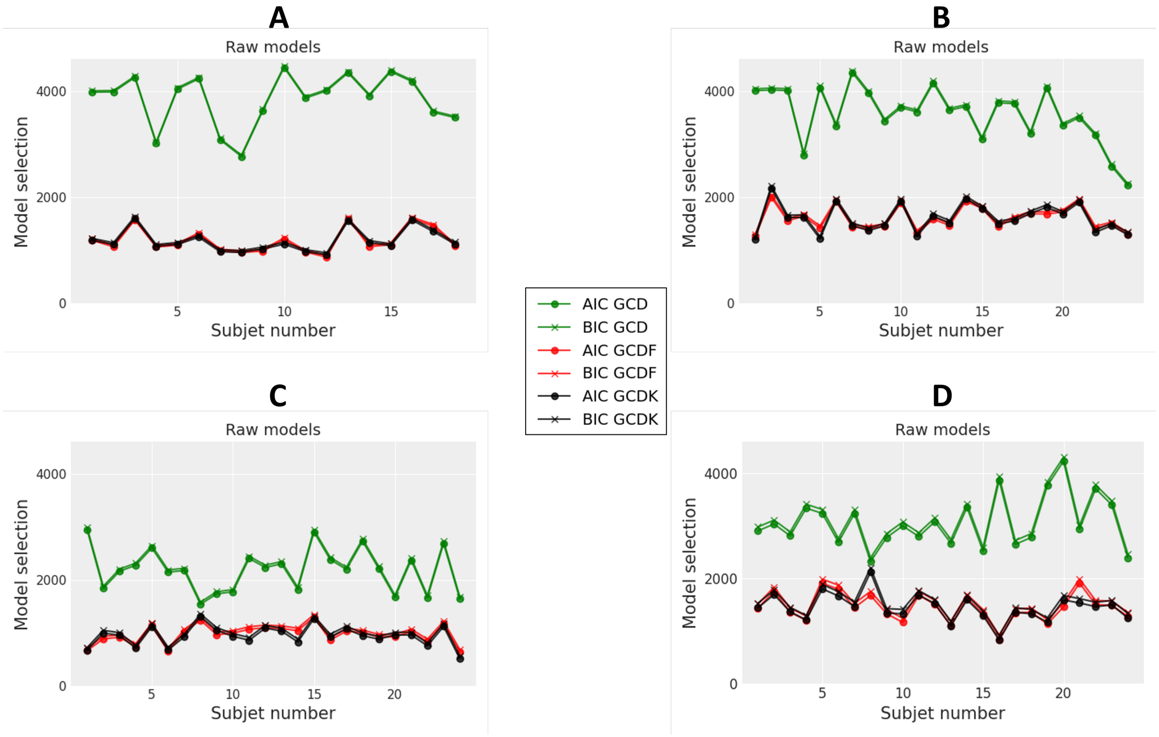

Note. Panels (A)–(D) correspond to Experiments 1–4. AIC = Akaike information criterion; BIC = Bayesian information criterion; GCD = gated cascade diffusion model; GCDF = gated cascade diffusion model with a filtering mechanism at the motor preparation level; GCDK = gated cascade diffusion model with a Kalman-Bucy filter at the motor preparation level. See the online article for the color version of this figure.

Table C1

Parameters From the Raw Models (GCD, GCDF, and GCDK) Averaged Across Subjects for Experiments 1–4

Exp	Model	v_1	v_2	v_3	v_4	v_5	k	dc	λ	λ'	ξ	g	r	Te_1	Te_2	Te_3	Te_4	Te_5	Tr	x_0
1	GCD						1.147					0.085	0.045	0.271					0.079	
1	GCDF						0.694		12.519			0.055	0.024	0.193					0.066	
1	GCDK						0.620			7.085	0.0013	0.045	0.019	0.084					0.050	
2	GCD	0.020						-0.026				0.058	0.049	0.293					0.089	0.004
2	GCDF	0.015						-0.016	35.937			0.039	0.032	0.267					0.084	0.002
2	GCDK	0.013						-0.014		20.408	0.0010	0.031	0.028	0.204					0.074	0.002
3	GCD	0.363	0.588	0.628	-0.594							0.259	0.070	0.188					0.067	-0.001
3	GCDF	0.083	0.155	0.223	-0.216				35.690			0.043	0.029	0.367					0.080	0.001
3	GCDK	0.042	0.105	0.154	-0.148					2.604	0.0028	0.011	0.012	0.225					0.046	0.001
4	GCD	0.438	0.472	0.534	0.583	-0.445						0.225	0.066	0.171	0.167	0.166	0.159	0.180	0.073	-0.006
4	GCDF	0.111	0.176	0.233	0.282	-0.180			52.543			0.043	0.034	0.337	0.330	0.323	0.305	0.369	0.084	-0.006
4	GCDK	0.102	0.169	0.209	0.246	-0.167				21.503	0.0017	0.034	0.028	0.266	0.265	0.250	0.230	0.311	0.072	-0.005

Note. Experiment 3 (recognition memory): subscripts 1-4 for parameter v correspond to conditions old words studied one time, old words studied two times, old words studied four times, and new words, respectively. Experiment 4 (lexical knowledge): subscripts 1-5 for parameters v and Te correspond to conditions very-low frequency words, low frequency words, medium frequency words, high frequency words, and pseudowords, respectively. GCD = gated cascade diffusion model; GCDF = gated cascade diffusion model with a filtering mechanism at the motor preparation level; GCDK = gated cascade diffusion model with a Kalman-Bucy filter at the motor preparation level.

(*Appendices continue*)

Appendix D
Additional Predictions From GCDF

Figure D1

Additional Predictions From GCDF With Varying Levels of Leak λ and Drift Rate v

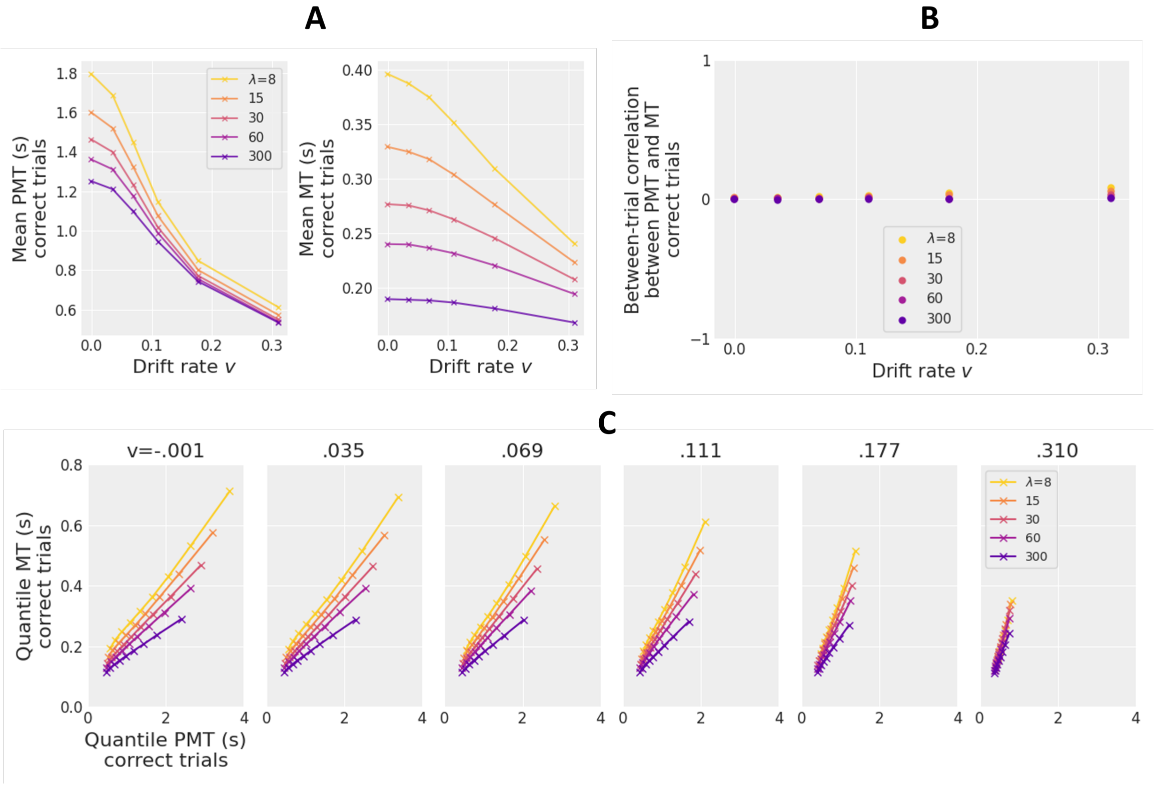

Note. Apart from the leak parameter, simulations used the best fitting GCD parameters averaged across subjects reported by Servant et al. (2021) and 100,000 simulated trials per condition. (A) Predicted mean PMT and mean MT in correct trials. (B) Predicted between-trial correlation between PMT and MT in correct trials. (C) Predicted PMT quantile–MT quantile plot in correct trials. GCD = gated cascade diffusion model; GCDF = gated cascade diffusion model with a filtering mechanism at the motor preparation level; PMT = premotor time; MT = motor time. See the online article for the color version of this figure.

Figure D2

Additional Predictions From GCDF With Varying Levels of Leak λ and Drift Rate v, and With Between-Trial Variability in Drift Rate (Normally Distributed With Mean v and Standard Deviation sv)

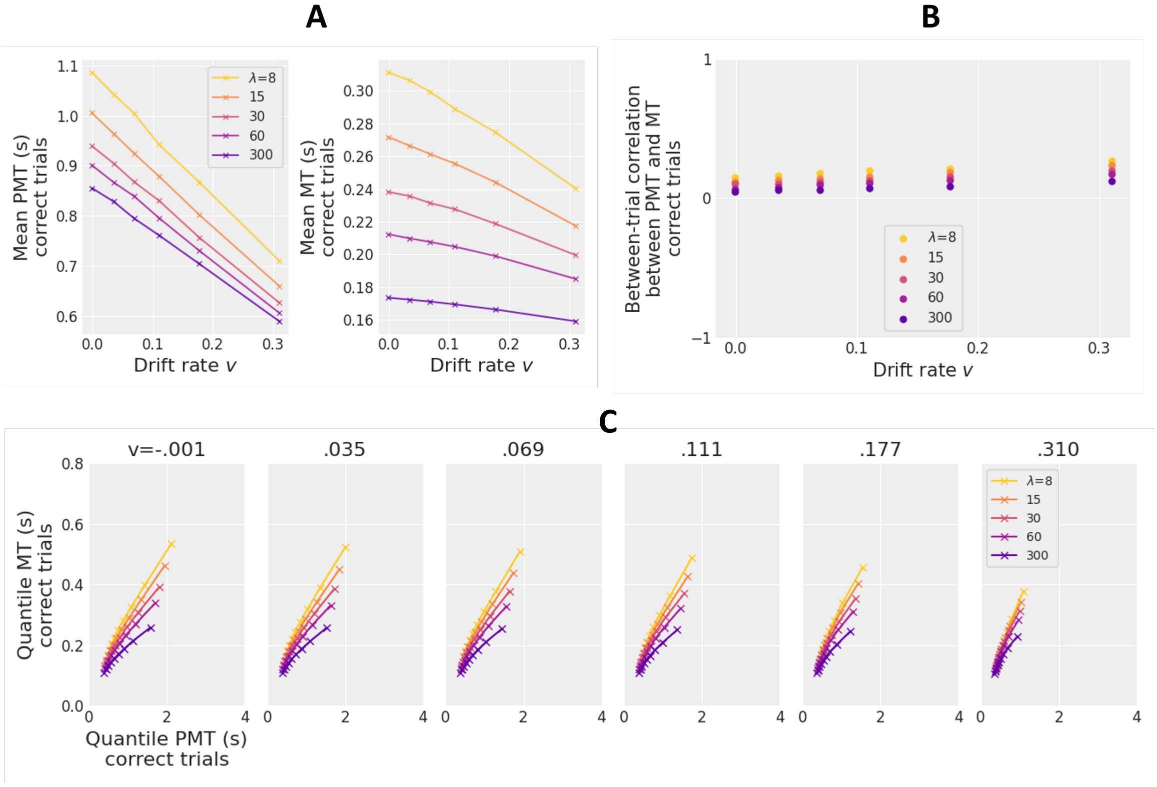

Note. Apart from the leak parameter, simulations used the best fitting GCD parameters averaged across subjects reported by Servant et al. (2021) and 100,000 simulated trials per condition. Parameter *sv* was fixed at .2. (A) Predicted mean PMT and mean MT in correct trials. (B) Predicted between-trial correlation between PMT and MT in correct trials. (C) Predicted PMT quantile–MT quantile plot in correct trials. GCD = gated cascade diffusion model; GCDF = gated cascade diffusion model with a filtering mechanism at the motor preparation level; PMT = premotor time; MT = motor time. See the online article for the color version of this figure.

(*Appendices continue*)

Appendix E
Methods for Experiments 2-4

Experiment 2

Participants

Manipulations of numerosity produce smaller modulations of mean RT compared to motion coherence manipulations. Consequently, we increased the sample size from 18 to 24 subjects in order to maintain a reasonable amount of statistical power while keeping electrophysiological and modeling work in manageable proportions.

Twenty-four students (five men; mean age: 20.6) from the University of Franche-Comté took part in the experiment in exchange for course credits or as volunteers. All subjects met the following criteria: being 18-30 years old, being right-handed, having a normal or corrected vision, and having no history of motor, psychiatric, or neurological disorder. Subjects were not aware of the purpose of the experiment and provided written consent to participate. This study was approved by the ethical committee for research of the university (Agreement No. CERUBFC-2022-01-18-002). It was not preregistered.

Apparatus

The experiment took place in a dimly lit room. Subjects sat on a comfortable chair at a distance of 75 cm from a 34.7 $\times$ 19.5 cm^2 liquid crystal display monitor (resolution: 1920 $\times$ 1080; framerate: 60 Hz). The experiment was programmed in Python, using functions from the PsychoPy library (Peirce et al., 2019). Response buttons were identical to those used in Experiment 1. Subjects' hands were faced palm-down, resting on a supportive cushion placed on their laps in order to minimize tonic muscular activity and maximize comfort.

Stimuli

For each trial, between 31 and 70 black dots were displayed on a gray background screen in random positions within a 10 x 10 virtual grid. Each dot was 0.24° in diameter. The horizontal and vertical separation between two adjacent dots (from center to center) was 1.15°.

Procedure

Participants were instructed to press the left button with their left thumb if they judged the number of dots displayed was less than or equal to 50, and the right button with their right thumb if they judged it was greater. Left responses to 31-50 dots and right responses to 51-70 dots were counted as correct. Participants were told not to count the dots but instead provide a global and rapid estimation of their number. Dots remained on the screen until the participant responded. An RT deadline was set to 4 s. If participants failed to respond by then, the message "Too late! Please respond faster." was displayed for 1.5 s. The intertrial interval was 1.5 s. Participants first completed a practice block of 40 trials, containing each of the 40 numerosity conditions presented in a random order. A feedback on performance ("correct response" or "incorrect response") was displayed for

1.5 s after each response. Practice trials were not considered in the analyses. Subjects then completed 30 blocks of 40 trials with a similar structure, except that no feedback was provided after each response. Blocks were separated by self-paced breaks. The experiment lasted about an hour.

EMG Recordings and Signal Processing

The procedure used for EMG recordings and signal processing was similar to Experiment 1, except that EMG signals were epoched -0.5 s to 4 s relative to stimulus onset. Trials with a high level of noise were discarded from analyses (2.7% of trials on average; range 0%-11.7%).

Models and Fit Procedure

The data were grouped into eight conditions represented by the mean number of dots N of each bin (33, 38, 43, 48, 53, 58, 63, 68). Following Ratcliff and McKoon (2018), we assumed that the drift rate v of GCD and GCDF is a linear function of N and the criterion (50):

$$v = dc + v_1(N - 50). \tag{E1}$$

Parameter v_1 accounts for interindividual differences in discrimination performance. Parameter dc (drift criterion) accounts for interindividual differences in the representation of the criterion. Without this parameter (i.e., assuming $dc = 0$), the representation of the criterion would correspond exactly to 50 dots for every subject, which is not realistic. Between-trial variability in drift rate is assumed to be normally distributed with mean v and standard deviation sv defined as follows:

$$sv = \eta_0 + \sigma_1\sqrt{N^2 + 50^2}. \tag{E2}$$

All parameters were fixed across numerosity conditions. We treated the starting point x_0 of the evidence accumulation process as a free parameter, resulting in seven (12) free parameters for the raw (full) GCD and eight (14) free parameters for the raw (full) GCDF.[8] All parameters (except dc and x_0) were constrained to be ≥ 0. Between-trial variability parameters sx_0, sTe, sTr, and $s\lambda$ were constrained to not exceed 180% of g, Te, Tr, and λ, respectively. Parameter sx_0 was further constrained to not exceed $2 * (g - |x_0|)$. Model selection statistics (AIC and BIC) were computed using Equations 11 and 12. By a two-sided binomial test, if 18 out of 24 subjects support one model over the other, then the result is significant.

Experiment 3

Participants

Twenty-four students (six men; mean age: 19.78) from the University of Franche-Comté took part in the experiment in exchange for course credits or as volunteers. All participants met the same inclusion criteria as for Experiment 2. Subjects were not aware of the

[8] Ratcliff and McKoon (2018) also evaluated a model variant in which subjects solve the task by comparing the number of dots to the number of blank spaces, and found similar fits. We found similar fits for this GCD and GCDF variant.

purpose of the experiment and provided written consent to participate. This study was approved by the ethical committee for research of the university (Agreement No. CERUBFC-2022-01-18-002). It was not preregistered.

Apparatus

The apparatus was identical to Experiment 2.

Stimuli

We selected a set of 1,058 French words (number of letters ranging from five to eight; $M = 6.50$; $SD = 1.12$) from the Lexique database (New et al., 2004). Word frequency ranged from one to six occurrences per million ($M = 2.96$, $SD = 1.14$). Words were presented in black against a gray background (font: Consolas) at the center of the screen. The height of the letters was 0.76°.

Procedure

The experiment consisted of 23 blocks (one training block with a feedback on accuracy after each trial and 22 experimental blocks without feedback), separated by self-paced breaks. Each block consisted of a study phase and a test phase. During the study, participants were instructed to learn 25 words. The first two words served as fillers to control for primacy effects, and the last two words served as fillers to control for recency effects. Among the 21 remaining words, seven were studied one time, seven were studied two times, and seven were studied four times. Words were presented in a random order, at a pace of 1 s. The test phase occurred right after the study phase and consisted of 42 words (21 old words and 21 new words, presented in a random order). Participants were instructed to press the left or the right button with their left or right thumb depending on whether the word was old or new (stimulus-response mapping balanced across participants). Each test word remained on screen until the participant responded, or until a 4 s RT deadline. If participants failed to respond by then, the message "Too late! Please respond faster." was displayed for 1.5 s. The intertrial interval was 1.5 s. Words presented in a block never appeared in another block (participants were made aware of this during task instructions). In addition, the assignment of words to blocks and conditions was randomly determined. The experiment lasted about an hour and a half.

EMG Recordings and Signal Processing

The procedure used for EMG recordings and signal processing was similar to Experiment 2. Trials with a high level of noise were discarded from analyses (12.4% of trials on average; range 1.6%-25%).

Models and Fit Procedure

Drift rate v and between-trial variability in drift rate sv were the only parameters free to vary between conditions. Similar to Experiment 2, we treated the starting point x_0 of the decision-making process as a free parameter, resulting in nine (16) parameters for the raw (full) GCD, and 10 (18) parameters for the raw (full) GCDF. All parameters (except drift rates and x_0) were constrained to be ≥ 0. Between-trial variability parameters sx_0,

sTe, sTr, and $s\lambda$ were constrained to not exceed 180% of g, Te, Tr, and λ, respectively. Parameter sx_0 was further constrained to not exceed $2 * (g - |x_0|)$.

Experiment 4

Participants

Twenty-four students (four men; mean age: 21.00) from the University of Franche-Comté took part in the experiment in exchange for course credits or as volunteers. All participants met the same criteria as for Experiments 2 and 3. Subjects were not aware of the purpose of the experiment and provided written consent to participate. This study was approved by the ethical committee for research of the university (Agreement No. CERUBFC-2022-01-18-002). It was not preregistered.

Apparatus

The apparatus was identical to Experiments 2 and 3.

Stimuli

Four lists of French words (5-12 letters) of differing frequencies (high frequency: range 10-2,736 occurrences per million; medium frequency: range 2-5; low frequency: range 0.5-1; very-low frequency: range 0.01-0.1) were created using the Lexique database (New et al., 2004). To obtain the stimuli used in the present experiment, the lists underwent the following steps. First, all plural and feminine agreements were removed. Second, low and very-low frequency words were screened by two students, and any words they did not know were eliminated. Third, a pool of 408 words was pseudorandomly selected from each list, with the constraint of obtaining a homogeneous number of letters across pools. For each pool, 204 words were randomly selected to become pseudowords (i.e., pronounceable nonwords), created with the multilingual pseudoword generator Wuggy (Keuleers and Brysbaert, 2010). Pseudowords were screened by two other students to ensure that they were pronounceable and did not correspond to an existing word. This procedure resulted in four lists, each list comprising 204 words (four for practice and 200 for the experiment) and 204 pseudowords (four for practice and 200 for the experiment). The four students recruited for screening stimuli did not participate in the experiment. Statistics relative to frequency and number of letters for the final sample of words used in the experiment are provided in Table E1. Words were presented in black against a gray background (font: Consolas) at the center of the screen. The height of the letters was 0.76°.

Procedure

Participants were instructed to press the left or the right button with their left or right thumb depending on whether the stimulus was a French word or not (stimulus-response mapping balanced across participants). Participants first performed a practice block of 32 trials (four words in each frequency level and 16 pseudowords) during which feedback on accuracy after each trial was provided and worked through 10 blocks of 160 trials (20 words in each frequency level and 80 pseudowords) with no feedback on accuracy. Blocks were separated by self-paced breaks. Each stimulus appeared once in the experiment.

Participants were thus exposed to the same word and pseudoword stimuli. Stimuli were randomly assigned to blocks and presented in a random order within blocks. Each trial started with the presentation of the stimulus until the participant responded, or until a 4 s RT deadline. If participants failed to respond by then, the message "Too late! Please respond faster." was displayed for 1.5 s. The intertrial interval was 1.5 s. Overall, the experiment lasted about an hour.

EMG Recordings and Signal Processing

The procedure used for EMG recordings and signal processing was similar to Experiments 2 and 3. Trials with a high level of noise were discarded from analyses (10.4% of trials on average; range 0.06%-27.3%).

Models and Fit Procedure

Drift rate (v), between-trial variability in drift rate (sv), and mean residual time added to predicted PMT (Te) were the only parameters free to vary between conditions. We treated the starting point x_0 of the evidence accumulation process as a free parameter, resulting in 14 (22) parameters for the raw (full) GCD and 15 (24) parameters for the raw (full) GCDF. All parameters (except drift rates and x_0) were constrained to be ≥ 0. Between-trial variability parameters sx_0, sTe, sTr, and $s\lambda$ were constrained to not exceed 180% of g, Te, Tr, and λ, respectively. Parameter sx_0 was further constrained to not exceed $2 * (g - |x_0|)$.

Table E1

Statistics Relative to Frequency and Number of Letters for the Sample of Words Used in Experiment 4

	Occurrences per million		Letters	
Word frequency condition	M	SD	M	SD
High frequency words	77.26	142.37	7.05	1.70
Medium frequency words	3.32	0.89	7.03	1.66
Low frequency words	0.72	0.15	7.05	1.70
Very low frequency words	0.04	0.03	7.05	1.69
